# Novel application of the published kinase inhibitor set to identify therapeutic targets and pathways in triple negative breast cancer subtypes

**DOI:** 10.1371/journal.pone.0177802

**Published:** 2017-08-03

**Authors:** Margarite D. Matossian, Steven Elliott, Van T. Hoang, Hope E. Burks, Theresa B. Phamduy, Douglas B. Chrisey, William J. Zuercher, David H. Drewry, Carrow Wells, Bridgette Collins-Burow, Matthew E. Burow

**Affiliations:** 1 Department of Medicine: Section of Hematology and Oncology, Tulane University, New Orleans, Louisiana, United States of America; 2 Department of Physics, Tulane University, New Orleans, Louisiana, United States of America; 3 Eshelman School of Pharmacy, University of North Carolina - Chapel Hill, Chapel Hill, North Carolina, United States of America; National Cancer Center, JAPAN

## Abstract

Triple negative breast cancers (TNBCs) have high recurrence and metastasis rates. Acquisition of a mesenchymal morphology and phenotype in addition to driving migration is a consequential process that promotes metastasis. Although some kinases are known to regulate a mesenchymal phenotype, the role for a substantial portion of the human kinome remains uncharacterized. Here we evaluated the Published Kinase Inhibitor Set (PKIS) and screened a panel of TNBC cell lines to evaluate the compounds’ effects on a mesenchymal phenotype. Our screen identified 36 hits representative of twelve kinase inhibitor chemotypes based on reversal of the mesenchymal cell morphology, which was then prioritized to twelve compounds based on gene expression and migratory behavior analyses. We selected the most active compound and confirmed mesenchymal reversal on transcript and protein levels with qRT-PCR and Western Blot. Finally, we utilized a kinase array to identify candidate kinases responsible for the EMT reversal. This investigation shows the novel application to identify previously unrecognized kinase pathways and targets in acquisition of a mesenchymal TNBC phenotype that warrant further investigation. Future studies will examine specific roles of the kinases in mechanisms responsible for acquisition of the mesenchymal and/or migratory phenotype.

## Introduction

Breast cancer, followed by lung and colorectal cancers, is the most prevalent neoplastic disease in women. Among U.S. women there are expected to be more than 290,000 new cases of invasive breast cancer in 2016 [1]. There has been minimal improvement in survival in patients with metastatic recurrent breast cancer over the past 30 years [2,3], highlighting the importance of discovering novel targeted therapies. Molecular subtypes of breast cancers are categorized based on the receptors they overexpress: Estrogen receptor (ER+), progesterone receptor (PR+), and EGFR receptor Her2 (HER2+) amplification. Effective therapies are currently available to target estrogen and HER2 receptors and provide benefit for the associated breast cancer subpopulations. Triple negative breast cancers (TNBCs) lack these targetable receptors, severely limiting therapeutic options and viable means for intervention. Phenotypically, TNBC frequently presents as a more advanced and metastatic disease than other breast cancer subtypes [4,5]. Acquisition of a mesenchymal phenotype, based on a change in cell morphology to more fibroblastic-appearing cells, upregulation of mesenchymal genes and downregulation of epithelial genes, alteration of the extracellular environment and promotion of a migratory behavior facilitates engagement of the cancer cells from the primary tumor, extravasation into the vasculature, and seeding in distal tissue sites to create metastatic lesions. One specific process that has been described in the acquisition of a mesenchymal phenotype, Epithelial-mesenchymal transition (EMT) is defined by the loss of individual cells’ epithelial genes (downregulation of E-cadherin) and upregulation of mesenchymal genes (vimentin, c-FOS, SNAI1, TWIST, ZEB1) and positively correlates with cancer cell progression to metastasis and therapeutic resistance [6–8]. A well-documented example of an EMT regulator is the mitogen-activated protein kinase (MAPK) family, which is activated by extrinsic cytokines and growth factor receptors. Once activated, MAPK members have diverse roles in maintaining the mesenchymal phenotype through interactions with other signaling pathways [9–11].

The mechanisms responsible for driving mesenchymal phenotypes and cell plasticity are complex and require regulation of various kinases and coordinated interactions of different signaling pathways. Kinases are key mediators of metastasis, growth and pro-proliferative pathways that are essential to tumor progression through regulation of mesenchymal genes and rearrangement of cytoskeletal components and EMT in a range of tumor types. In fact, kinases regulate some aspects of mesenchymal phenotypes (upregulate mesenchymal gene expressions and promote migration) but may not necessarily directly regulate another aspect (downregulate epithelial genes). Kinases often require coordination with other regulatory proteins to drive metastasis. Unregulated kinase activity affects many cellular pathways involved in tumorigenesis [12,13], and targeting these kinases has been a primary focus of both past and emerging therapeutic development studies. To date, the kinases known to contribute to tumor progression and metastasis represent only a small sample of the expansive human kinome, which is composed of 518 kinases [14]. Furthermore, deep sequencing reveals 375 kinases (approximately 75%) are expressed in various breast cancer cell lines [15]. A large proportion of the human kinome have uncharacterized functions [16]. Our laboratory recognizes that therapeutically important and innovative targets could emerge from this subset of currently understudied and poorly understood kinases. Kinases are tractable targets for drug discovery, and the discovery of new kinase targets useful for treatment of the receptor negative and more aggressive breast carcinomas is an important goal.

Recently, broad screen approaches using small molecule inhibitor or RNAi libraries have been employed to implicate alternative pathways and individual kinases involved in acquisition of a mesenchymal cellular phenotype by cytoskeletal remodeling [17,18] and EMT reversal, also known as mesenchymal-to-epithelial transition (MET) [19–22]. We utilized the Published Kinase Inhibitor Set (PKIS), a library of 906 compounds provided by GlaxoSmithKline (GSK) consisting of compounds from a wide range of chemical scaffolds that were originally prepared to target known kinase therapeutic targets (including polo-like kinase, c-Jun N-terminal kinase, protein kinase B, insulin-like growth factor, and others); this library set is composed of two combined libraries, PKIS1 and PKIS2 [23]. Much of the set has been profiled against a large panel of kinases, and thus the use of the set in a phenotypic screen allows interrogation of previously unidentified or understudied kinase pathways [24]. To identify additional kinase pathways that play a role in regulating the mesenchymal phenotype in TNBC through genetic alterations, or promotion of a mesenchymal morphology and migratory behavior, we utilized this small molecule kinase inhibitor library to screen three TNBC cell lines with mesenchymal phenotypes: MDA-MB-231, MDA-MB-157 and BT549, looking for compounds that induced reversal to more epithelial-like morphologies.

## Materials and methods

### Reagents

Dulbecco’s modified Eagle’s medium (DMEM), Dulbecco’s phosphate-buffered saline (DPBS), phenol-red free DMEM, fetal bovine serum (FBS), minimal essential amino acids (MEMAA), non-essential amino acids (NEAA), antibiotic/anti-mitotic penicillin/streptomycin (pen/strep), sodium pyruvate, L-glutamine, trypsin/EDTA, trypan blue stain (0.4%) and ethylenediaminetetraacetic acid (EDTA 0.5 M, pH8) were obtained from GIBCO (Invitrogen; Carlsbad CA). Insulin was purchased from Sigma-Aldrich (St. Louis MO) and charcoal stripped (CS) FBS from HyClone (Thermo Scientific; Logan UT). Dimethyl sulfoxide (DMSO) was obtained from Research Organics, Inc (Cleveland OH).

### Cell culture

Human MDA-MB-231, BT549 and MDA-MB-157 cells were obtained from the American Type Culture Collection (ATCC, Manassas, VA, USA) and are characterized as triple-negative/basal B mammary carcinoma. Cells were maintained in DMEM supplemented with 10% FBS, non-essential amino acids (NEAA), MEM amino acids, anti-anti (100 U/mL), sodium pyruvate and porcine insulin (1 x 10^−10^ mol/L) at 37°C in humidified 5% CO_2_. For treatment studies, cells were grown in phenol red-free DMEM supplemented with 5% charcoal-stripped FBS and supplemented with NEAA, MEM amino acids, Gluta-Max and penicillin (100 U/mL).

### The published kinase inhibitor sets (PKIS)

The PKIS is openly available in screening quantities from the SGC-UNC. Instructions for requesting PKIS can be found at www.sgc-unc.org and chemical structures and other pharmacologic activity for the compounds can be found at https://www.ebi.ac.uk/chembldb/extra/PKIS/compounds.html. The set is typically provided as 1 μL of a 10 mM solution in DMSO, dispensed in 384-well plates. A material transfer agreement was created to ensure that the screening results are made publicly available. Larger aliquots of requested compounds were delivered as solids, dissolved in DMSO to a 1 mM stock solution, and stored at -20°C. The solutions were diluted in culture media and used at 1 μM concentrations, as determined by dose-response studies.

### Crystal violet staining

MDA-MB-231, BT549 and MDA-MB-157 cells were plated at 5,000 cells per well in 96-well TC plate in 5% charcoal stripped (CS) DMEM. After 48 hours of exposing the cells to CS DMEM media, cells were treated with the vehicle or selected GSK library compounds for 72 hours and the plate was incubated in 37°C, 5% CO_2_. The plate was then harvested by adding glutaraldehyde (10 μL) to each well for 20 minutes. After rinsing and drying the plate, the cells were stained with 0.1% crystal violet in 90% methanol (50 μM) for 20 minutes. After another rinse, the cells were left overnight to dry, and the following day morphological alterations of the cells were visualized with an inverted microscope and images were recorded. Cells were lysed with 33% acetic acid and quantified to determine proliferation after treatment.

### RNA isolation and quantitative real time PCR

MDA-MB-231 cells were plated in 5% CS DMEM and pre-treated for 72 hours with DMSO (control), or PKIS compounds (1 μM). Cells were harvested and total RNA was isolated using RNeasy (Qiagen, Valencia, CA) following manufacturer’s protocol, and the quantity and quality determined by absorbance (260, 280 nm). Total RNA (2 μg) was reverse-transcribed (iScript kit, BioRad Laboratories, Hercules, CA) and analyzed by qRT-PCR. Primer sequences are as follows (Invitrogen, Carlsbad, CA): *β-actin* F—5’- GGCACCCAGCACAATGAAGA-3’; *β-actin* R-5’- ACTCCTGCTTGCTGATCCAC -3’; *CDH1* F-5’-AGGTGACAGAGCCTCTGGATAGA-3’, *CDH1* R-3’-TGGATGACACAGCGTGAGAGA-3’. *VIM* F-5’-GAGAACTTTGCCGTTGAAG -3’; *VIM* R-5’- GCTTCCTGTAGGTGGCAATC-3’, *SNAI1* F-5’-ACCACTATGCCGCGCTCTT-3’; *SNAI1* R-5’- GGTCGTAGGGCTGCTGGAA-3’, *ZEB1* F-5’-ACACAAGCGAGAGGATCATG-3’; *ZEB1* R-5’- CGGAATCTGAATTTGCTTCTACC-3’, *c-FOS* F-5’-CCTGTCAAGAGCATCAGCAG-3’, *c-FOS* R-5’-GTCAGAGGAAGGCTCATTGC-3’. Quantitative reverse transcription-PCR was conducted as previously published [25]. Data represented as normalized fold expression compared with DMSO control of biological triplicate samples ± SEM.

### Transwell migration assay

Migration assays were performed following the manufacturer’s instructions (BD Biosciences, San Jose, CA, USA). MDA-MB-231 cells were grown in charcoal stripped DMEM (5% FBS) for 48h, then pretreated for 72h (1 μM) with DMSO, GSK198271, GSK448459, GSK346294, and GSK1173862. Transwell inserts (8 μm pore size; BD biosciences; San Jose, CA) were placed into each well containing 1 mL 10% FBS DMEM media. Pretreated cells in opti-MEM suspension were placed on each insert (500 μL, 25,000 cells per well). After 24 hours, membranes were scrubbed to remove non-migrated cells and membranes were removed and mounted on glass slides. Migrated cells were visualized by microscopy and counted. Data is represented as number of migrated cells per field of view for triplicate experiments.

### Immunofluorescence staining

PKIS- or DMSO-treated MDA-MB-231 cells were fixed in formalin (10% buffered formalin phosphate, Fischer Scientific, Hampton NH) and permeabilized with Triton-X100 (MP Biomedicals, St. Ana CA). Cytoskeletal components were stained with AlexaFluor 555-conjugated antibody against phalloidin (Cell Signaling, clone 8953, 1:200, Danvers MA). Cells were counterstained with DAPI (NucBlue Fixed Cell Stain ReadyProbe, Life Technologies, Carlsbad CA). ApoTome (commercial structure illumination microscopy by Zeiss, Thornwood, NY) fluorescent images were captured on an inverted microscope (Zeiss) and digitally filtered to obtain optical slices.

### Image-based morphometric analysis

Polygonal outline and length measurement tools provided in the AxioVision software (Zeiss) formed the basis for morphometric analysis. Based on information obtained from these tools, four metrics for cellular morphology were identified and defined (see below). Aspect ratio was determined through the use of the length measurement tool, and two perpendicular length measurements were reported. Cellular area coverage, nucleus: cytoplasm area ratio, and circularity assessments were determined from area and perimeter measurements obtained by the polygonal outline tool (Table 1). A total of 45 individual cells were measured from each treatment group, including the DMSO control. Selection criteria for cells included: 1) well-defined border (eliminates most cells in aggregates or dimly stained cells) and 2) must contain only one nucleus (eliminates dividing cells and cells out of the plane of focus).

**Table 1:** Cellular morphology quantification calculations. Calculations and measurements are described with the associated formulas based on cellular dimensions of individual cells.

Metric	Definition	Mathematical formula
Aspect ratio	length of the longest axis divided by longest length of cell in orthogonal axis	$AR=\frac{L_{long}}{L_{short}}$
Area coverage	surface area covered per cell, as outlined by cell membrane	$\frac{A}{cell}$
Nucleus to cytoplasm ratio	ratio of area covered by nucleus to cytoplasm, as outlined by membrane	$\frac{A_{nucleus}}{A}$
Circularity	ratio of area to perimeter, as outlined by cell membrane	$c=4\pi \left(\frac{A}{P^{2}}\right)$

### Microscopy imaging

The Nikon eclipse TE2000-s inverted fluorescence microscope and camera with x-cite series 120 illuminator (Nikon; Melville, NY), in conjunction with IP Lab version 3.7 software (Rockville, MD) were used in the visualization of crystal violet-treated cells to observe morphological changes.

### Western blot

Western blot analyses were conducted as published^25^. After 24 hours of treatment in 10% fetal bovine serum DMEM medium, membranes were probed with primary CDH1 antibody (Cell Signaling Technology, 24E10) according to manufacturer’s protocol. IR-tagged secondary antibodies were purchased from LiCor Biosciences (1:10000). Blots were analyzed by the Odyssey Infrared Imaging System (LiCor Biosciences). Experiments were conducted in triplicate.

### Patient-derived xenografts

Tissues were obtained through the Louisiana Cancer Research Consortium Biospecimen Core and were processed in compliance with NIH regulations and institutional guidelines, and approved by the institutional review board at Tulane University. Animals were anesthetized (isofluorane) prior to the tumor cell implantation and survival surgery and monitored post-anesthesia till ambulatory. Post-operative monitoring of injection and incision sites were daily until healed. Animals were treated with anesthesia (CO2 inhalation) prior to euthanasia/cardiac puncture, which is the only procedure (besides tumor implantation and survival surgery) involving discomfort, distress or pain that warrants anesthesia. Animals were given meloxicam (20mg/kg/day) for 72 hours’ post-surgery for pain. Animals were euthanized by administration of CO2 anesthesia prior to cervical dislocation at the end of all experimental procedures, when tumor volume exceeded 1000mm3. If we observed ulceration or necrosis at tumor injection or implantation sites, mice were euthanized. These procedures are consistent with the recommendations of the Panel on Euthanasia of the American Veterinary Medical Association.: Animal procedures will be carried out in full accordance with established standards set forth in the Guide for the Care and Use of Laboratory Animals (NIH publication No. 85–23). Procedures involving chronic conditions or treatment of laboratory animals were performed using sterile equipment (syringes, needles, surgical instruments) and aseptic technique. Due to their immunocompromised status, mice were housed in sterile cages (5/cage) and provided with autoclaved food and water. Mice were observed daily for signs of illness (e.g., reduced activity or water intake) or cutaneous ulceration, and tumors were measured weekly during tumor initiation. This project was approved by Tulane University Institutional Animal Care and Use Committee (IACUC). *SCID/Beige* (CB17.Cg-*Prkdc*^*scid*^*Lyst* ^*bg*^/Crl) were used. The autosomal recessive SCID (Prkdc^scid^) mutation results in severe combined immunodeficiency affecting both the B and T lymphocytes. The autosomal recessive beige (Lyst^bg^) mutation results in defective natural killer (NK) cells. We must use animals with compromised immune systems in order to prevent rejection of the xenografted human tumors. A triple-negative lumpectomy sample of approximately 15mm in length was excised from the patient after informed consent. Tumor tissues from each patient were cut into 1 x 1 mm pieces under aseptic sterile conditions, coated with full factor matrigel (Cat No. 354234, Fisher Scientific, Waltham, MA, USA) and implanted bilaterally into the mammary fat pads (m.f.p.) of SCID/beige mice. Tumors were measured using a digital caliper after ostensible tumor take was established. Tumors were passaged when tumor volume grew to 750–1000 mm^3^. To passage, mice were euthanized by CO_2_, tumors were removed and sliced with a scalpel to 3 x 3 mm pieces, coated in full factor matrigel, then implanted bilaterally into the m.f.p. of new SCID/beige mice that were anesthetized using a mix of isoflurane and oxygen delivered by mask. After surgery, mice were given meloxicam (20 mg/kg/day, 3 days post-surgery) for pain. For PCR analyses, 3 x 3 mm tumor explants were plated in 12-well plates with 2 ml of 10% DMEM and treated for 72 hours with DMSO (control) or GSK198271A at 1 μM. After treatment, tumor pieces were collected, and RNA was extracted using QIAzol Lysis Reagent (Cat No. 79306; Qiagen, Valencia CA). Synthesis of cDNA and qRT-PCR was performed as described previously.

### Kinase assay

The kinase assay was performed by Reaction Biology Corporation (Malvern, PA, USA) and contains 350 kinases of interest, as described previously [26].

### Statistical analysis

Studies run in triplicate were analyzed by unpaired Student’s *t*-test (Graph Pad Prism V.4). *p*-Values < 0.05 were considered statistically significant. Raw data from the figures, as well as statistical analyses, are available in S7 Fig.

## Results

### Small molecule inhibitors induced epithelial morphologies in phenotypically mesenchymal cell lines

**Fig 1:**
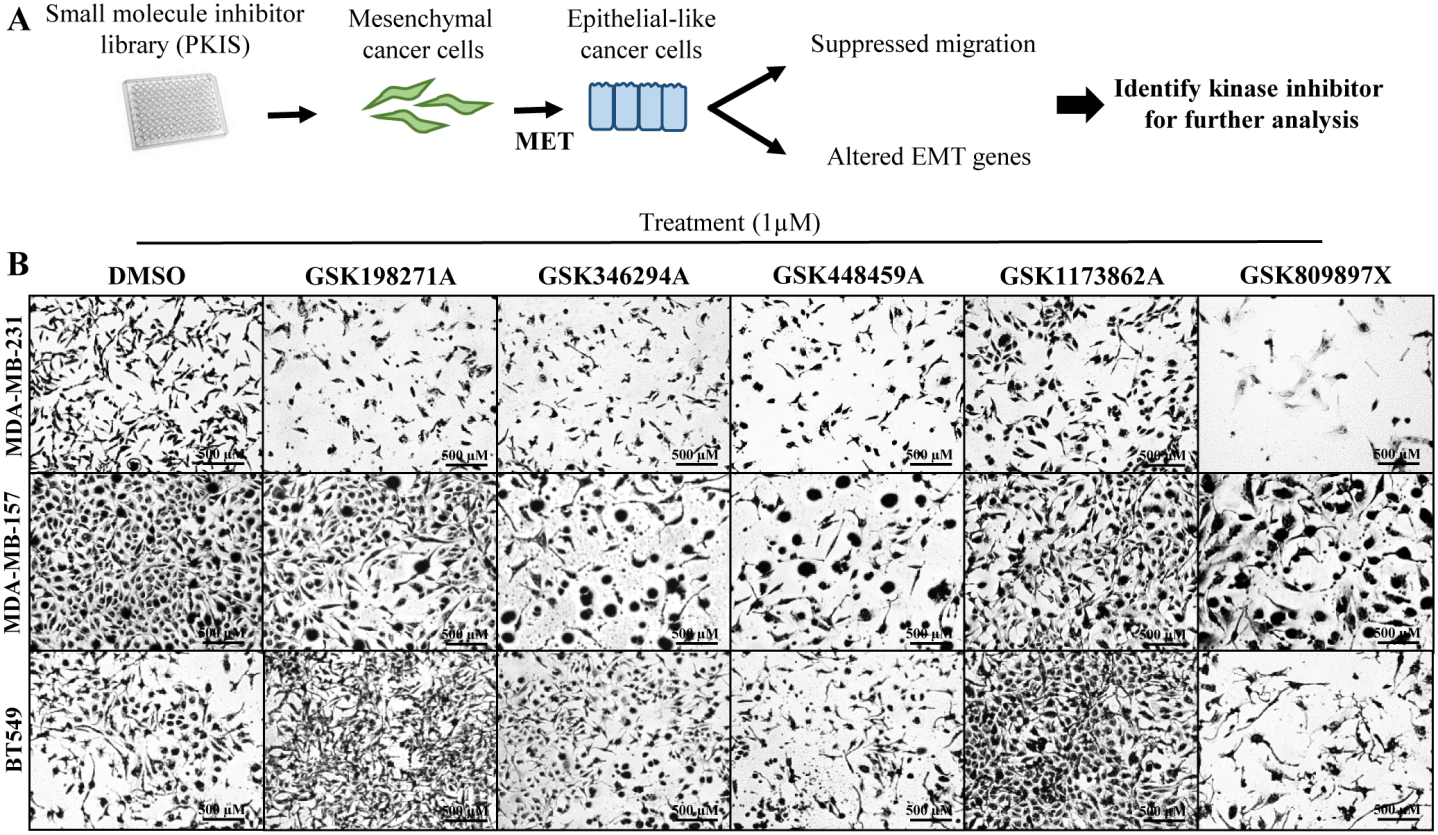
Cell morphology screen of small molecule kinase inhibitors from PKIS library induce MET in mesenchymal breast cancer cell lines. (A) Three phenotypically mesenchymal breast cancer cell lines (MDA-MB-231, MDA-MB-157 and BT549) were treated with small molecule kinase inhibitors provided in the GlaxoSmithKline PKIS library or vehicle (DMSO) for 72 hours’ treatment (1μM). Schematic representation of the broad screen approach. (B) Crystal violet staining techniques were used to observe changes in cellular morphology after treatment. Images were captured with Nikon Camera at 100X magnification. Representative inset images of select hit compounds are shown; additional hit compounds as well as full images are found in S1 Fig. LBH-589 treatment (100 nM) was used as a positive control [27]. Morphological changes to a more epithelial phenotype included rounder cells, cells that congregate (indicative of increased cell-cell contacts) and fewer fibroblastic-like cells.

Driven by the need to identify treatment options to target TNBC, an aggressive breast cancer subtype, we treated the TNBC cell lines MDA-MB-231, MDA-MB-157 and BT549 with the PKIS library for 72 hours (Fig 1A). We decided to do our initial screen based on morphology differences, because a change in cell morphology to an epithelial phenotype is a process central to many aspects of a mesenchymal phenotype, due to downregulation of mesenchymal genes, upregulation of epithelial genes, and/or promotion of a migratory phenotype due to cytoskeletal rearrangements. Once we identified small molecule inhibitors that altered the mesenchymal morphology, we then screened for other components that define a mesenchymal phenotype. Cell morphology was observed through crystal violet stain and images were captured for each treatment via brightfield microscopy (Fig 1B). Small molecule inhibitors that visually altered cell morphologies were recorded following treatment. Cells consistent with an acquired epithelial phenotype were identified through observance of a rounded cell morphology and growth in close proximity to surrounding cells with increased cell-cell contacts, and a greater cytoplasm: nuclear ratio. Cells designated as maintaining a mesenchymal phenotype were fibroblast-like in appearance, exhibited a loss of polarity, and possessed long, thin processes (consistent with invadopodia).

Our initial screen in MDA-MB-231 cells identified 36 compounds that reversed EMT based on cellular morphology observations. The initial screen was performed blind: the primary kinase targets of the small molecule inhibitor hits were unknown at the time of treatments and active compounds were selected solely based on cellular morphology observations. To confirm that these observed changes were not cell type specific, we next treated other TNBC (MDA-MB-157, BT549) cell lines with the PKIS library (Fig 1B, S1 Table). Overall, we identified twelve compounds that consistently reversed the mesenchymal phenotype in all phenotypically mesenchymal TNBC cell lines. These compounds represent a number of different chemical classes and were originally published as inhibitors of a range of kinase targets including IGF-1R, PLK1, EGFR, VEGFR, PDGFβ, LCK and AKT (S1 Table). These 12 compounds were selected for further analysis. To assure that the observed changes in the morphology screen were unique to TNBC cells, we screened the PKIS library with a luminal, epithelial non-invasive cell line, MCF-7 (S1 Fig).

### Identification of small molecule inhibitors that induced MET and/or suppressed cell migration

Acquisition of epithelial markers and suppression of cellular motility are defining characteristics of the reversal of a mesenchymal phenotype. In some specific processes, this is known as mesenchymal-to-epithelial transition, MET. These events can be preceded by cytoskeletal rearrangements and observed changes in cellular morphology [9,28]. To confirm if the observed morphological effects on MDA-MB-231 cells were related to MET on a gene expression level, qRT-PCR was performed. Upregulation of E-cadherin (CDH1) indicates reversal of a mesenchymal phenotype to an epithelial phenotype. Concordantly, downregulation of mesenchymal proteins, including transcription factors c-FOS, ZEB1, SNAI1 and the cytoskeleton protein vimentin (VIM) indicates reversal of a mesenchymal phenotype as well. (S2 Fig). We followed-up our morphology screen with the following molecular and biological experiments first using the MDA-MB-231 cell line.

**Fig 2:**
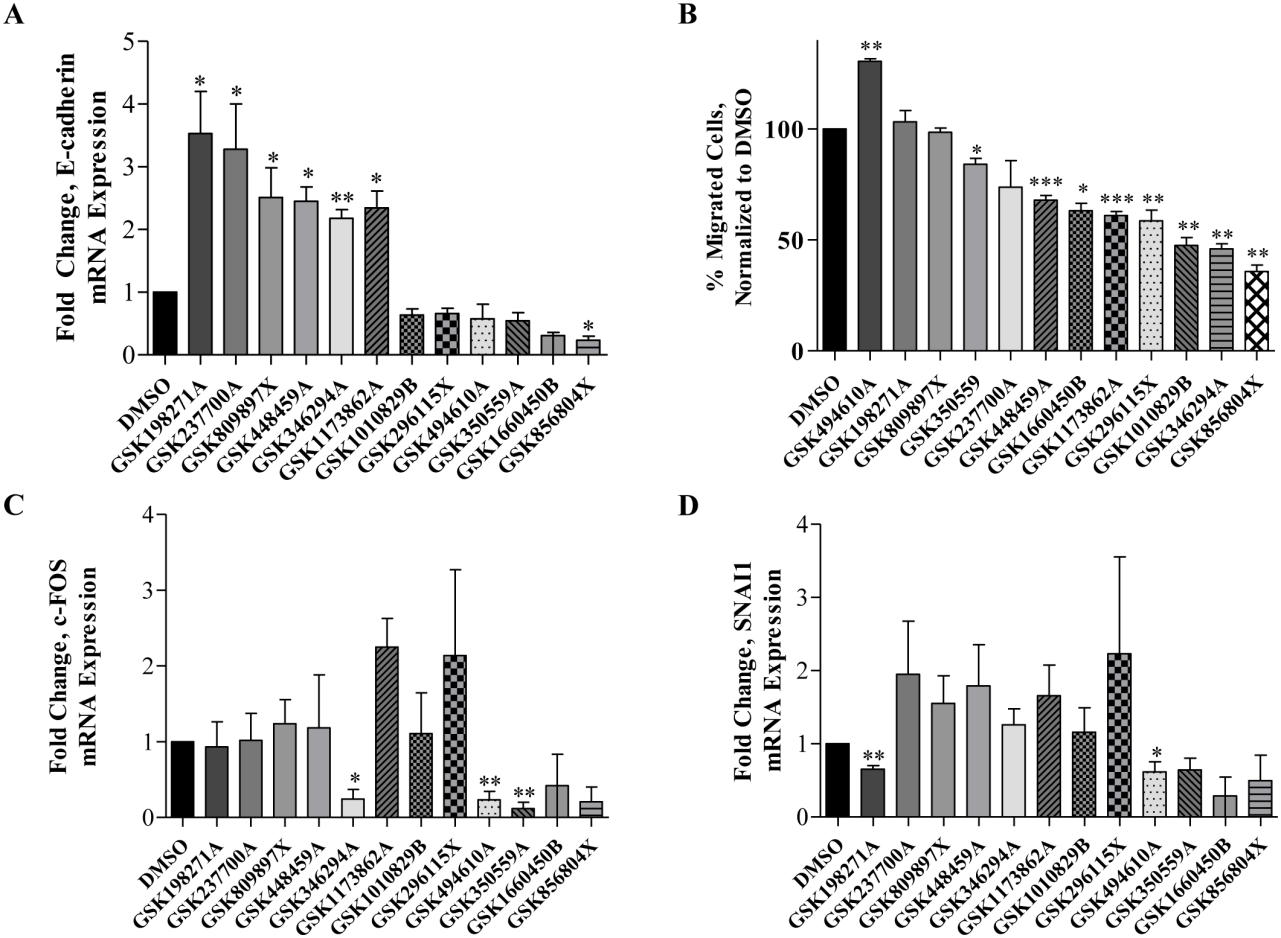
Kinase inhibition induces MET through enhanced MET gene expression and suppression of cellular migration. (A) Fold change in the epithelial marker CDH1 was observed after MDA-MB-231 cells were treated with 1 μM of select small molecule inhibitors after 72 hours’ pretreatment. Data was obtained by qRT-PCR and normalized both to actin and the vehicle (DMSO)-treated control designated as 1. (B) Transwell migration assays of MDA-MB-231 cells revealed GSK346294A, GSK1010829B and GSK1173862A most significantly suppressed TNBC cellular migration. Migrated cell counts were normalized to vehicle. Images of migrated cells were captured with microscopy at 200X. Cells were counted in eight views of the wells, and migration was performed in triplicate for each treatment. (* p < 0.05, *** p < 0.001). (C, D) Mesenchymal transcription factors c-FOS and SNAI1were also analyzed was observed after MDA-MB-231 cells were treated with 1 μM of select small molecule inhibitors after 72 hours’ pretreatment. Data was obtained by qRT-PCR and normalized both to actin and the vehicle (DMSO)-treated control designated as 1. For all experiments, N = 3, error bars represent SEM and significantly different * p < 0.05, *** p < 0.001.

In MDA-MB-231 cells, six of the twelve tested kinase inhibitors increased CDH1 expression (Fig 2A). Two kinase inhibitors significantly increased CDH1 expression, including the pyrazolopyridazine GSK198271A that was previously identified as active in a phenotypic anti-malarial screen and a thiophene benzimidazole, GSK346294A, originally prepared to target PLK1. Other compounds that increased CDH1 include pyrrolopyrimidine GSK1173862A originally prepared to target IGF-1R, two additional thiophene benzimidazoles originally prepared to target PLK1, GSK237700A and GSK448459A, and the diaminopyrimidine GW809897X which was originally prepared to target VEGFR2. Interestingly, an opposite effect on CDH1 expression occurred after treatment with GSK494610A, GSK1010829B, GSK296115X, GSK350559A, GSK1660450B and GW856804X.

Suppression of cellular migration is another defining aspect of EMT reversal; next, we performed transwell migrations using MDA-MB-231 cells after 72-hour pre-treatment with the twelve kinase inhibitor hits (Fig 2B). Treatment with DMSO was utilized as the vehicle control (100% migration). After treatments, PKIS compounds with lower migration percentages compared to DMSO control indicate the compounds suppressed cell migration. Seven of the twelve compounds significantly reduced cell migration compared to DMSO control: GW856804X, GSK346294A, GSK1010829B, GSK296115X, GSK1173862A, GSK1660450B and GSK448459A. Notably, some of the inhibitors that did not increase CDH1 expression suppressed migration (GW856804X, GSK296115X, and GSK1660450B). Notably, the top compound that showed the most significant changes in promoting an epithelial phenotype based on the gene expression analyses, GSK198271A, did not affect cellular migration.

On a transcriptional level, we examined gene changes of c-FOS, and SNAI1, transcription factors that promote a mesenchymal phenotype (Fig 2C and 2D). Treatment of MDA-MB-231 cells with GSK198271A, the EGFR-targeting compound that increased CDH1 expression most significantly, also reduced SNAI1 expression significantly, but had no effect on c-FOS mRNA expression. GSK346294A, a PLK1-targeting compound, and GSK1173862A, an IGF1-R targeting compound, that also significantly increased CDH1 expression, significantly reduced c-FOS expression, but had no effect on SNAI1 expressions. Other compounds that increased CDH1 expression significantly (GSK23700A, GSK809897X, GSK448459A) did not significantly downregulate mesenchymal gene expression of SNAI1 nor c-FOS. Additionally, some PKIS compounds that did not increase CDH1 expression reduced expression of the mesenchymal markers. GSK494610A and GSK350559A, EGFR-targeting compounds, are such examples because they reduce c-FOS and SNAI1 expressions.

**Fig 3:**
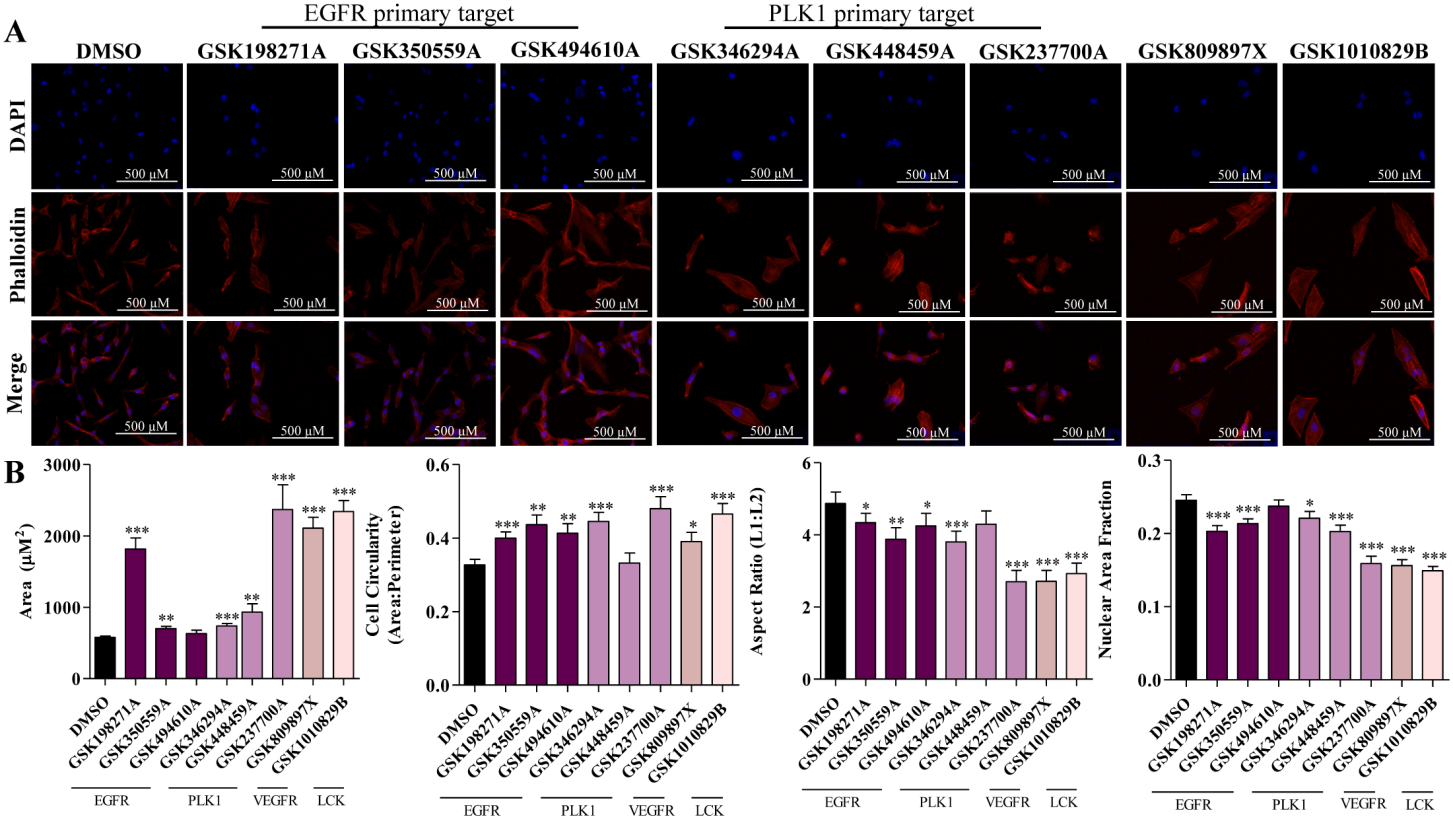
Immunofluorescence staining to observe morphological changes after treatment with GSK inhibitors. (A) Apotome-based microscopic imaging of triple negative MDA-MB-231 cells treated with DMSO (control; panel *a*), GSK198271A, GSK350559A, GSK494610A, GSK346294A, GSK448459A, GSK237700A, GSK809897X, and GSK1010829B. The samples were pretreated for 72 hours before staining and experiment was performed in triplicate. A phalloidin stain was used to highlight actin filaments (red), DAPI stained for the nucleus (blue). (B) Quantitative morphological analysis of MDA-MB-231 cells after treatment with select GSK compounds. A minimum of 45 cells were quantified per treatment group. Measurements of cellular morphology after immunofluorescence staining and Apotome microscopy imaging are reported. Area is defined as the length of the cell x the width; Circularity = 4(π) x (area/perimeter^2^). Aspect Ratio = length/width; Nuclear Area Fraction = (Area of Nucleus/Area of Cell). Greater area, cell circularity, aspect ratio and nuclear area fraction are associated with a mesenchymal phenotype due to cytoskeletal rearrangement [31]. For quantification experiments, N ≥ 45, error bars represent SEM and significantly different * p < 0.05, *** p < 0.001.

Inhibitors that most profoundly affected the mesenchymal phenotype, either due to acquisition of epithelial markers, downregulation of mesenchymal genes or due to suppression of cell migration, were selected for further morphometric analyses to quantify observed morphology changes in MDA-MB-231 cells. Quantification involved a series of measurements including aspect ratio analysis, which refers to the overall dimensions of the individual cells. First cells were stained with phalloidin, to highlight actin cytoskeletal filaments, and DAPI, a nuclear stain. Immunofluorescence was performed to visualize the morphologies and confocal microscopy was employed to capture the images (Fig 3A). Dimensional analyses include nuclear area fraction (nuclear: cytoplasmic ratio), circularity of the cell, overall area and aspect ratio. GSK237700A treatment increased overall cellular area and circularity most significantly, followed by GSK198271A and GSK1010829B. GSK809897X treatment led to the lowest aspect ratio, a measurement that examines the longest length related to the shortest length of the cells and is reflective of cellular polarity. GSK237700A and GSK1010829B also had reduced aspect ratios. Finally, GSK198271A, GSK1010829B and GW809897X exhibited the smallest nuclear area fraction. A larger nuclear area in relation to cytoplasm is a feature often found in phenotypically mesenchymal cells [29,30]. Thus, a smaller nuclear area fraction is reflective of an epithelial phenotype. Overall, GSK198271A treatment of MDA-MB-231 cells most dramatically reversed mesenchymal morphologies (Fig 3B).

**Fig 4:**
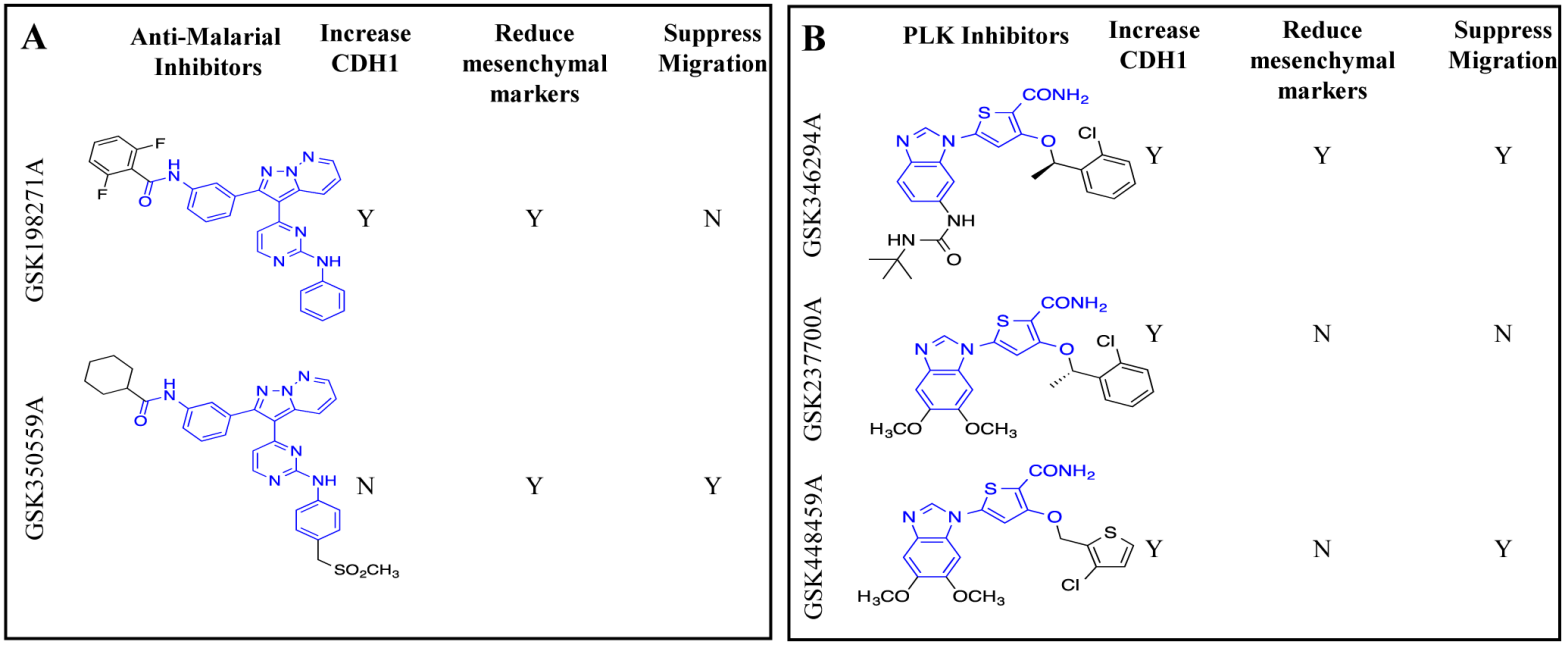
Analysis of structurally similar PLK inhibitors and anti-malarial inhibitors. (A) Three small molecule PLK inhibitors (GSK346294A, GSK448459A and GSK237700A) are similar in structure and have similar effects on reversal of a mesenchymal phenotype as indicated by an increase in CDH1 and suppression of cellular migration. (B) Two structurally similar anti-malarial inhibitors (GSK198271A, GSK350559A) identified in the initial screen alter cellular morphology, but only GSK198271A increases CDH1 expression. These data indicate there is a potential off-target effect that drives the observed effects of the inhibitors. Pharmacophores are outlined in blue. Compounds selected for further in-depth analyses altered the mesenchymal phenotype by affecting gene expressions or suppression of cellular migration.

The PKIS can be subdivided into groups of compounds with common core structure. In some cases, compounds with common core structures had divergent effects on morphology changes in breast cancer cell lines. For example, GSK198271A and GSK350559A, have highly similar structures, but had opposing effects on CDH1 and VIM expression (Fig 4A). The PKIS is comprised of inhibitors that function via competitive binding with the cofactor ATP. Because the ATP binding pocket is conserved but not identical across the kinome, many of the PKIS compounds have some degree of promiscuity in their kinase activity. Consequently, the kinase for which a compound was originally prepared might not be the only potently inhibited kinase, and importantly the original kinase may not be a contributor to the phenotype resulting from compound treatment. Furthermore, three structurally similar PLK inhibitors in the thiophene benzimidazole chemotype increased CDH1 and suppressed cellular migration, albeit to varying degrees (Fig 4B). Based on these observations, we hypothesize that there is a common off-target kinase or kinase family that is inhibited by the three PLK inhibitors (GSK346294A, GSK448459A, GSK237700A). Analysis of the structures reveals the importance that the structure itself may have in on- or off-target activity of the PKIS compounds and how this affects reversal of the mesenchymal phenotype.

### GSK198271A promotes MET and novel potential MET kinase targets and pathways are identified

**Fig 5:**
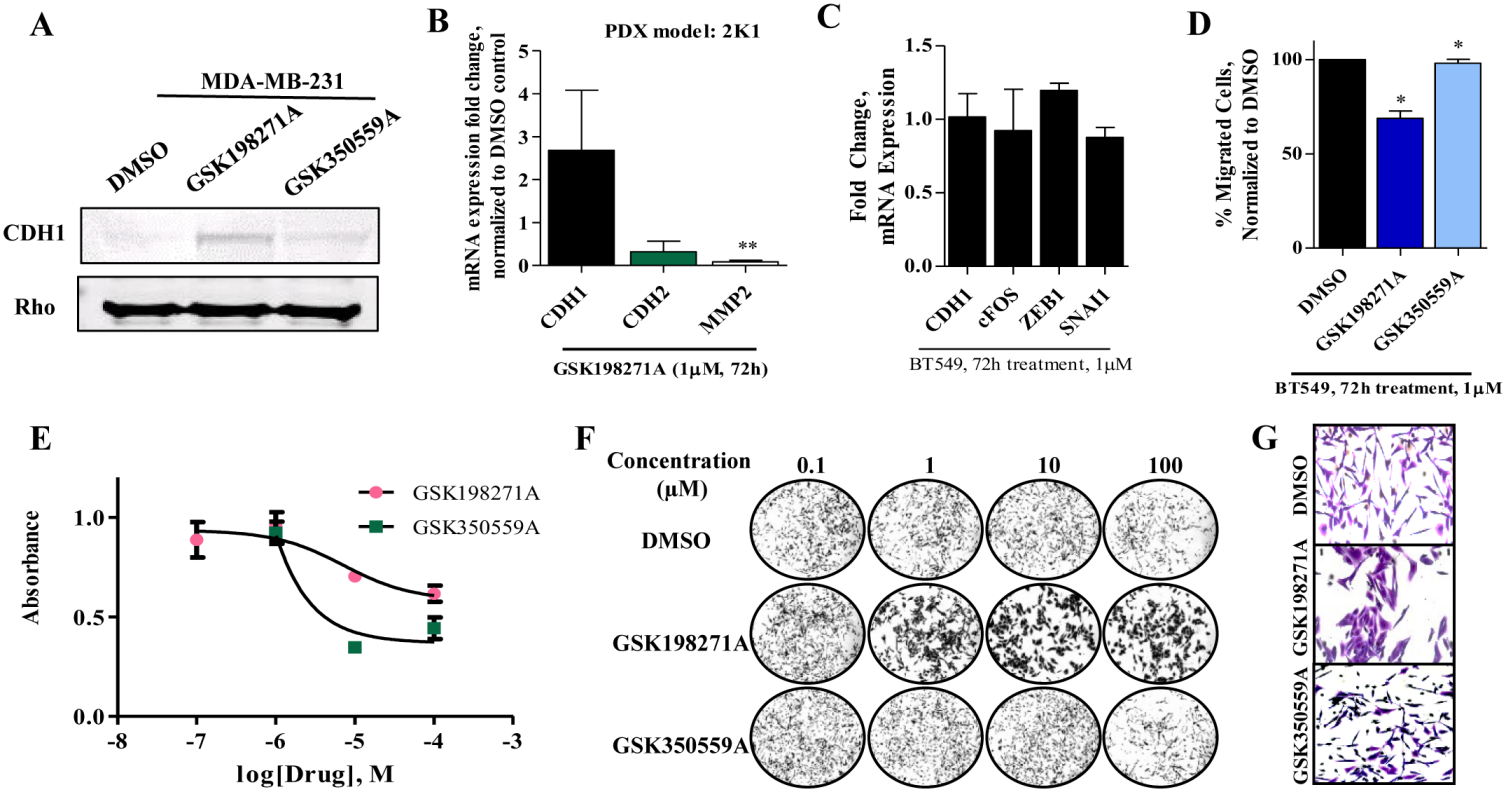
The pyrazolopyridazine GSK198271A promotes MET. (A) Western blot analyses of MDA-MB-231 cells after treatment with GSK198271 confirms CDH1 protein expression in increased. Full length western blots are found in S3 Fig. Cells were pre-treated for 72 hours at 1μM; data was normalized to Rho. (B) Treatment of PDX tumors ex vivo with GSK198271 resulted in increased the epithelial marker CDH1 mRNA expression and reduced expression of CDH2 and MMP2, mesenchymal markers. Tumor pieces were treated for 72 hours at 1μM. (C) GSK198271A and not GSK350559A suppresses migration in another TNBC cell line, BT549. (D) Neither GSK198271A nor GSK350559A significantly increase CDH1 expression in BT549 cells. (E) GSK198271A and GSK350559A affected cellular proliferation at drug concentrations greater than 1μM. MDA-MB-231 cells were treated for 72 hours, then crystal violet stained and imaged. Absorbance was measured at 570 nm to obtain dose-response curves. Data was normalized to DMSO and performed in triplicate. (F,G) Representative images are enlarged for 1μM concentrations. Images were taken at 200x magnification using the Nikon color microscope. For qRT-PCR experiments, N = 3, error bars represent SEM and significantly different * p < 0.05, *** p < 0.001.

After establishing the application of the PKIS library to identify novel kinase pathways involved in EMT reversal, we focused on the small molecule inhibitor that had the most dramatic effects on EMT gene expressions, GSK198271A. The following data compares the gene expression and behavioral profiles of two small molecule inhibitors that share similar pharmacophore, but have differing effects on acquisition of an epithelial phenotype based on the initial screening results, GSK198271A and GSK350559A. First, we confirmed increased expression of CDH1 after GSK198271A treatment at a protein level using western blot of MDA-MB-231 cells. Rho was visualized for normalization. We observed significant overexpression of CDH1 protein in GSK198271A treated cells, compared to DMSO (Fig 5A). Interestingly, we did not observe these effects in GSK350559A treated cells, confirming our previously acquired PCR data. Next we utilized a TNBC patient-derived xenograft model in an *ex vivo* experiment to confirm GSK198271A treatment promotes an epithelial phenotype on a gene expression level in a translational model. Tumor pieces were treated for 72 hours in triplicate with GSK198271A and DMSO control. Similar to data analyzed from MDA-MB-231-treated cell lines, GSK198271A increased epithelial marker (represented by CDH1) mRNA expression and reduced mesenchymal marker (represented by CDH2 and matrix metalloproteinase 2, MMP2) expressions (Fig 5B). To evaluate if the results were cell line specific, we repeated the migration assay and analysis of CDH1 mRNA expression in another triple negative cell line, BT549. Although neither GSK198271A nor GSK350559A increased CDH1 expression (data not shown), GSK198271A reduced c-FOS and SNAI1 expressions, although not significantly (Fig 5C). Furthermore, GSK198271A did suppress cell migration (Fig 5D).

**Fig 6:**
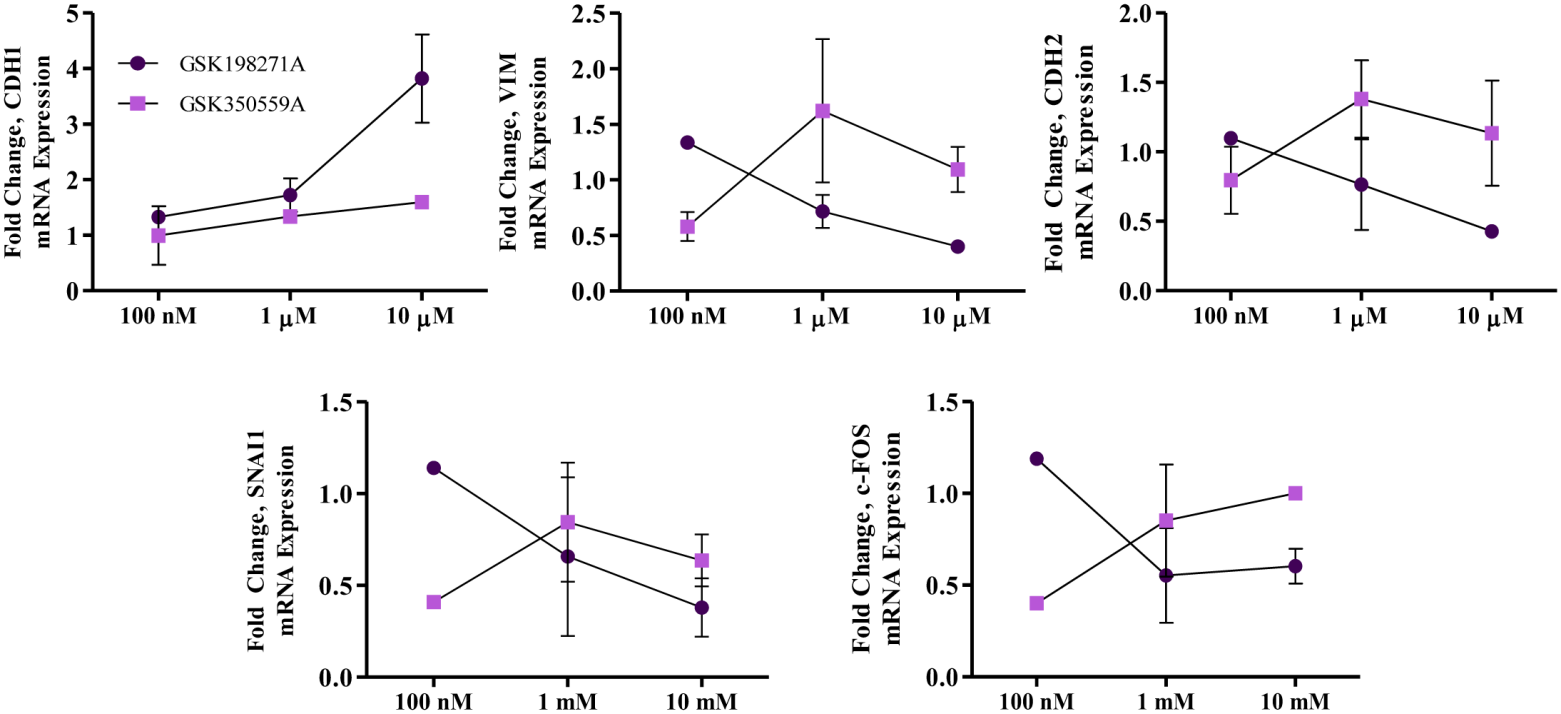
Kinase assay identified off-target kinase activities of GSK198271A and GSK350559A. Dose-response analyses of various mesenchymal-associated genes comparing GSK198271A to GSK350559A. qRT-PCR was performed to analyze mRNA expression of CDH1, an epithelial marker, and mesenchymal proteins and transcription factors (VIM, CDH2, c-FOS, SNAI1). For all experiments, N = 3, error bars represent SEM and significantly different * p < 0.05, *** p < 0.001.

Next we next sought to determine the effects of the pyrazolopyridazine kinase inhibitors (GSK198271A, GSK350559A) on cell proliferation and viability in a dose dependent manner. Cell viability was determined with crystal violet analysis and demonstrates loss of cellular survival with increasing drug concentrations (Fig 5E–5G). GSK350559A suppressed cell proliferation at concentrations greater than 1μM; GSK198271A also suppressed cell proliferation at this concentration, although not as dramatically as GSK350559A. To evaluate a potential polypharmacologic effect that is responsible for the observed genetic and behavioral differences between the two pharmacologically-similar compounds, we analyzed mesenchymal gene expressions with varying doses (100nM, 1μM, 10μM). GSK198271A, and not GSK350559A, increased CDH1 and reduced VIM, c-FOS CDH2 and SNAI1 expressions in a dose-dependent manner (Fig 6A).

Because the compounds are structurally similar, but have distinct effects on changes to both cell morphology and genomic expression, as GSK198271A mostly upregulates epithelial and suppresses mesenchymal genes and GSK350559A suppresses the migratory abilities of the cells, we were interested in determining if these differences were due to an off-target kinase unique to GSK198271A. We utilized a broad kinase screen provided by Reaction Biology Corporation to identify targets inhibited by both pyrazolopyridazines (Table 2). We divided the kinases into targets common to both inhibitors, and targets more unique to GSK198271A. Targets more unique to GSK198271A included HER2, TAOK2, and members of the SRC family kinases, HCK, LYN, LCK, CSK and c-SRC. This data suggests that the SRC pathway potentially plays a role in driving a mesenchymal phenotype, although future studies are required to investigate this in greater depth. In TCGA data provided by the Cancer Genome Atlas, we find HCK, LYN, LCK, and CSK are higher in basal-like breast cancer subtypes (S4 Fig). TAOK2 appears to have lower expression in basal-like subtypes (S4 Fig), although Kaplan-Meier survival analyses show higher expression of TAOK2 is associated with reduced overall survival in a cohort of TNBC patients (HR of 3.06, n = 68 patients) compared to non-TNBC patients in the same study (HR = 0.67, n = 526 patients; S5 Fig) [32,33]. Both TAOK2 and HCK are overexpressed in breast cancers, based on patient data provided by cBioPortal (S5 Fig) [34,35]. Another candidate target was inhibited at varying degrees by some of the top compounds of interest, STK10, also known as LOK (S1 Fig). We show relative expressions of TAOK2, HCK and STK10 in different breast cancer cell lines in S6 Fig. Data was obtained using data from the Gene expression based Outcome for Breast cancer Online algorithm (GOBO), a web-based analysis tool that utilizes Affymetrics gene expression data [36].

**Table 2:** Common targets and unique targets of GSK198271A and GSK350559A. Unique targets of GSK198271A include EGFR, HER2, TAOK2, and the SRC family kinases HCK, LYN, LCK, CSK and c-SRC. Data is shown as percent activity, or the remaining activity of the kinase in the presence of the inhibitor.

Common Targets (% Activity)			Unique Targets (% Activity)		
	*GSK198271A*	*GSK350559A*		*GSK198271A*	*GSK350559A*
DDR1	-0.5	0.5	EGFR	0	42
FGR	0	1.5	HER2	0	25
YES1	0.5	1	TAOK2	0.5	40.5
HER4	1	1	HCK	1	22
BRK	0.5	5	LYN	2	13
BMX	4	6	LCK	2.5	11
RET	4	8	CSK	4	17
TNIK	4.5	4	c-SRC	4	26

## Discussion

Despite emerging small molecule therapeutic development, the TNBC subtype remains elusive to available targeted chemotherapies and requires discovery of novel targetable pathways and proteins involved in the pathogenesis of this aggressive subtype. The mesenchymal characteristics of TNBCs promotes a metastatic phenotype; specifically, altered morphology with accompanying genomic changes from expression of epithelial genes to expression of mesenchymal genes and acquisition of migratory behavior contributes to TNBC cancer cells’ ability escape the primary tumor and metastasize. The maintenance of an epithelial phenotype is critical in the prevention of tumor cell migration and invasion, enhancing patient survival and overall prognosis [37,38]. Although some kinases have been extensively investigated as regulators of key cell signaling processes that promote a mesenchymal phenotype and cell motility, a substantial number of kinases have not yet been characterized to determine if they play a role in these processes. To address this, we utilized a commercially available small molecule inhibitor library in a morphology screen of various TNBC cell lines to identify novel pathways and potential targetable kinases in invasive breast cancer subtypes. Future studies will examine the specific roles the identified kinases have in acquisition and maintenance of a mesenchymal morphology and characteristics and/or cell motility.

Twelve small molecule inhibitors representing various kinase pathways from PKIS1 and PKIS2 libraries reverted TNBC cells to an epithelial phenotype based on morphology. Of those twelve inhibitors, eleven kinase inhibitors were ultimately identified to exhibit the potential to reverse mesenchymal phenotype, based on the acquisition of epithelial markers and subsequent loss of mesenchymal markers (GSK198271A, GW809897X), solely based on suppression of TNBC migration (GSK1660450B, GW856804X, GW296115X), or a combination of migration inhibition and epithelial phenotype acquisition (GSK346294A, GSK1173862A, GSK448459A and GSK237700A). Only GSK494610A did not promote a mesenchymal nor migratory phenotype in the follow-up studies. Crystal violet staining for morphology, qRT-PCR analyses, transwell migration studies and quantitative morphometric analyses provided evidence of the altered phenotype, after the initial morphology screen. Of the ‘hit’ kinase inhibitors identified, two were originally identified in an antimalarial phenotypic screen as EGFR inhibitors (GSK198271A, GSK350559A), one was originally prepared to inhibit IGF-1R (GSK1173862A), and the remainder originated in drug discovery programs targeting PLK1 (GSK346294A, GSK237700A GSK448459A), VEGFR2 (GW809897X), AKT (GSK296115X) and LCK (GSK1010829B).

Interestingly, because each of these compounds elicited diverse changes in molecular and biological studies, this suggests that the kinase inhibitory profiles of the individual kinases have differential effects on promoting a mesenchymal phenotype. After our initial morphology screen, we were interested in identifying kinases that altered morphology through a mesenchymal-epithelial transition (upregulation of epithelial markers, suppression of mesenchymal markers); we then used CDH1 to further screen the selected compounds. We discovered that some kinase inhibitors that changed the cells’ morphologies did not upregulate CDH1. These data suggest that these inhibitors affect cell morphology and mesenchymal phenotype through a CDH1-independent mechanism, and not necessarily through MET. Furthermore, the differences in the assays between structurally related compound that have the same primary target suggests that specific kinases differentially inhibited by one compound and not the structurally similar compound may be responsible for the observed phenotypic changes. For example, GSK237700A, GSK448459A and GSK346294A were originally designed to inhibit PLK1 and have similar structures, but have varying effects on cell morphology, mesenchymal and epithelial gene expressions and migratory behavior. This is also evident when comparing GSK198271A activity with GSK350559A, both originally published as EGFR kinase inhibitors showing activity in an anti-malarial phenotypic screen. These two compounds share a common pharmacophore, but again the changes of substituents on the core greatly affects the activity of the compounds.

We chose to focus this study on evaluating the differences between GSK198271A and GSK350559A to demonstrate the valuable tool this screen provides to identify novel kinase targets and pathways, although other hit compounds will be evaluated in future publications. Additionally, the observed changes in morphology may differ between cell lines, due to the variety of molecular TNBC subtypes that exist as well as the fact that immortalized cell lines contain varying mutations that may affect gene expression changes. To address this, we utilized a translational *ex vivo* patient-derived xenograft model to show that the EMT gene expression effects seen with GSK198271A treatment was not limited to immortalized cell lines. Furthermore, CDH1 protein overexpression was confirmed in treatment with MDA-MB-231 cells treated with GSK198271A, but not with GSK350559A. We also examined the effect of GSK198271A and GSK3505559A treatments on cell proliferation. We did not observe significant inhibition of cell proliferation at the concentrations utilized in our studies; however, after staining with crystal violet we observed a much more dramatic change in cell morphology after GSK198271A treatment in MDA-MB-231 cells. These data further support our hypothesis that there is a kinase target and/or family in GSK198271A driving the MET changes, and this target is more specific to GSK198271A than GSK350559A. To elucidate a potential target, we utilized a large kinome enzymatic screen and discovered a number of kinase candidates. Future studies will parse out which of these identified kinases are capable of promoting a mesenchymal phenotype in epithelial, non-invasive cell lines. Two of these candidate kinases, TAOK2 and HCK, have not been previously investigated as regulators of EMT and acquisition of cell motility/invasion in breast cancer, although one group recently referenced TAOK2 as a potential activator of TNBC cell growth [39]. Furthermore, members of the SFK family, including HCK, have been investigated as regulators of cell migration and motility in cancers [40,41], although the specific role of HCK in acquisition of a motile phenotype in metastatic breast cancers has not yet been investigated. Finally, we found another potential kinase target that was inhibited, at varying degrees, by multiple ‘hit’ compounds in our screen, STK10. STK10 is additionally upregulated in basal B TNBC subtypes, which represent a more mesenchymal phenotype. Interestingly, a recently published study that also screened the PKIS library also identified STK10 as a novel kinase target, albeit as a candidate target that modulates cancer cell growth and angiogenesis [23]. These findings from this study encourages us to pursue STK10 in future characterization studies.

We understand EMT and acquisition of a metastatic phenotype is a complicated process defined by not one single mechanism, but various mechanisms that interplay. Thus, we found it difficult to claim that one compound of interest was more interesting than another solely because it altered one aspect of acquisition of a mesenchymal phenotype (cell motility) and not another (gene expression changes). Based on this, as we gathered data from the cell morphology screen and subsequent molecular and biological analyses, we determined we could not use a set criteria to rank the compounds. However, this also makes our screening data valuable, in that we can focus future investigations of novel kinase pathways to the specific mesenchymal and/or migratory component that was altered in this screen. It is also important to note that the compounds available in the PKIS library are not selective inhibitors of single kinases. Rather, the compounds inhibit multiple kinases at a range of potency in values. Consequently, the kinase responsible for eliciting the observed morphological changes is not always immediately evident from the initial screening. Furthermore, we understand that there might be a poly-pharmacology effects responsible for the observed conversion to an epithelial phenotype. Consistent with this, patient survival data supports that overexpression of cassettes of kinases may in fact be responsible for promoting cancer cell progression and metastasis. Future studies to define the individual and combinatorial role for the identified kinases in development of a mesenchymal phenotype of cancer cells would potentially lead to second generation PKIS structure based compounds with focused selective targeting activity. A limitation with our qRT-PCR analyses in the screen is the use of actin as a house-keeping gene; because we utilized phalloidin to highlight cytoskeletal components in our morphometric quantification, it would be more appropriate to follow-up studies with specific kinase targets using a different house-keeping gene such as GAPDH.

Elucidating novel pathways and kinases that promote a mesenchymal phenotype in TNBC would identify drug targets for an aggressive subtype of breast cancer that has limited treatment options to date. Our primary objective was to evaluate the novel application of the PKIS library as a tool to identify previously uninvestigated kinase pathways and potential targets involved in TNBC. The major translational impact of this project lies in the potential for discovery of novel targeted therapies designed to prevent metastatic lesions that are the primary cause of mortality in breast cancer patients. This discovery would drastically change patient survival outlook and lead to a previously unknown molecular pathway that has wide therapeutic application in neoplasias independent of breast cancer. The primary objective of our small molecule inhibitor screen was to identify key kinase pathways and previously uncharacterized kinases that may have key regulatory roles in EMT and acquisition of a mesenchymal and invasive phenotype.

## Supporting information

S1 Table Top twelve small molecule inhibitors from the PKIS library that affect cellular morphology in TNBC cell lines. Images were captured at 100x magnification. Inhibitor names and structures are provided, as well as a list of top targets of the compounds.
(DOCX)

S1 Fig Cell morphology screen of PKIS library with the control epithelial cell line, MCF-7. Only GSK809897X appeared to affect the luminal, epithelial non-invasive cell line MCF-7 cell morphologies in addition to triple negative breast cancer cell lines utilized in the cell morphology screen. Images were captured at 100x magnification.
(DOCX)

S2 Fig PKIS compounds affected mesenchymal gene expression. Fold change in the mesenchymal protein VIM (S2A) and transcription factors ZEB1 (S2B) was observed after MDA-MB-231 cells were treated with 1 μM of select small molecule inhibitors after 72 hours’ pretreatment. Data was obtained by qRT-PCR and normalized both to actin and the vehicle (DMSO)-treated control designated as 1. N = 3, error bars represent SEM and significantly different * p < 0.05, *** p < 0.001.
(DOCX)

S3 Fig Example of full-length western blots images of MDA-MB-231 cells treated with DMSO (control, lane 1), GSK198271 (1 μM, 24 hours, lane 2) and GSK350559 (1 μM, 24 hours, lane 3). E-cadherin molecular weight is 135 kDa (S3A). Rho was utilized to normalize (35 kDa; S3B).
(DOCX)

S4 Fig The Cancer Gene Atlas (TCGA) gene expression data shows HCK, TAOK2, LYN, LCK CSK and c-SRC relative gene expression by RNAseq in breast cancer subtypes Luminal A, Luminal B, HER2-enriched and Basal Like. Red indicates upregulation, blue indicates downregulation.
(DOCX)

S5 Fig TAOK2 and HCK expressions in breast cancers. (S5A) High TAOK2 expression is associated with reduced overall survival is a cohort of TNBC-positive patients (n = 68) compared to TNBC-negative patients (n = 526). Hazard ratio was calculated to be 3.06 with a significant p-value of 0.0299. Data provided by the PROGgene database [32,33]. (S5B) TAOK2 and (S5C) HCK are amplified in breast cancers (amplification = red, mutation = green), according to patient data provided by cBioPortal [34,35].
(DOCX)

S6 Fig STK10, HCK and TAOK2 relative expressions in breast cancer cell lines, divided by subtype. STK10, and not HCK nor TAOK2, is upregulated in basal B TNBC molecular subtypes. Expression data is provided online and represents the various breast cancer subtypes and was obtained by the Gene expression-based Outcome for Breast cancer Online (GOBO; S6A) [36]. Cell line specific data for STK10, HCK and TAOK2. The various breast cancer cell lines commonly used are shown (S6B). Cell lines are grouped based on the TNBC molecular subtype into which they are categorized: Red indicates basal A subtype, grey indicates basal B subtype and blue indicates luminal subtypes.
(DOCX)

S7 Fig Complete data and statistical analyses. This supplementary spreadsheet contains complete data of individual data points, with accompanying statistical analyses, and is organized by the order of the figures as they appear in the manuscript.
(XLSX)
