# Supplementary material for: Novel application of the published kinase inhibitor set to identify therapeutic targets and pathways in triple negative breast cancer subtypes
Source: PLoS One. 2017 Aug 3;12(8):e0177802. doi: 10.1371/journal.pone.0177802 (PMC5542472; doi:10.1371/journal.pone.0177802)
Supplement: S1 Table — Images were captured at 100x magnification. Inhibitor names and structures are provided, as well as a list of top targets of the compounds. (DOCX) [file pone.0177802.s001.docx]

**S1 Table.** Top twelve small molecule inhibitors from the PKIS library that affect cellular morphology in TNBC cell lines. Inhibitor names and structures are provided, as well as a list of top targets of the compounds.

|  | **Notes** | **BT549** | **MDA-MB-157** | **MDA-MB-231** |
| --- | --- | --- | --- | --- |
| **Blank Control** |  | **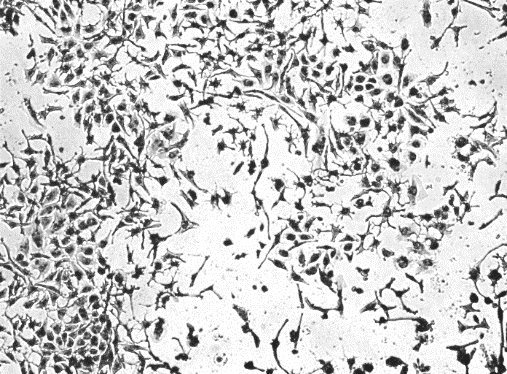**  **500 µM** | **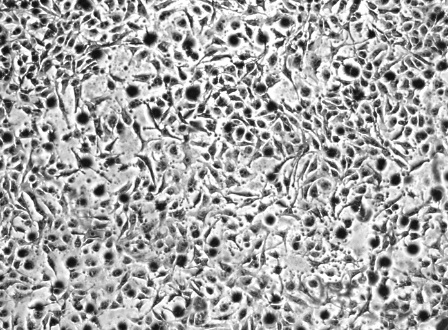**  **500 µM** | **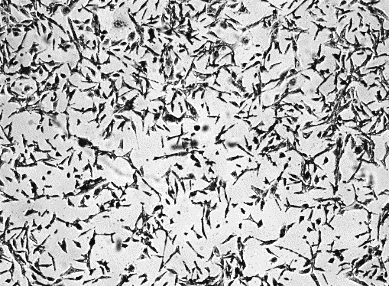**  **500 µM** |
| **GSK237700A**   | **Benzimidazole_N-thiophene series**  **@ 100 nM, 2 kinases > 50% I**  **Top targets: PLK1 90%, LOK/STK10 54%** | **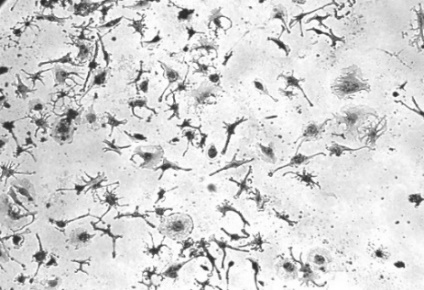**  **500 µM** | **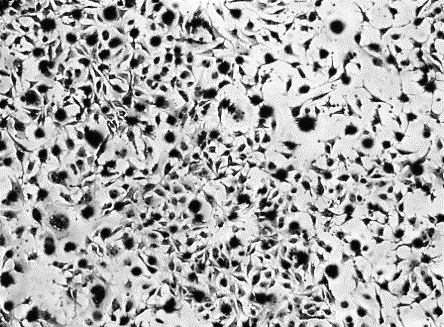**  **500 µM** | **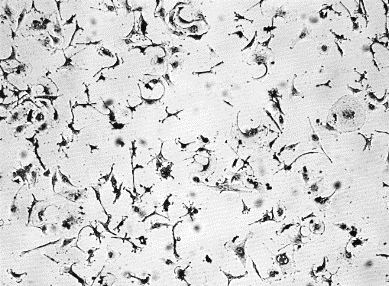**  **500 µM** |
| **GSK448459A**   | **Benzimidazole_N-thiophene series** | **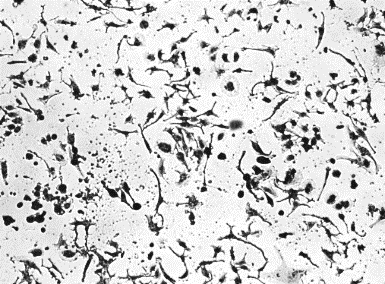**  **500 µM** | **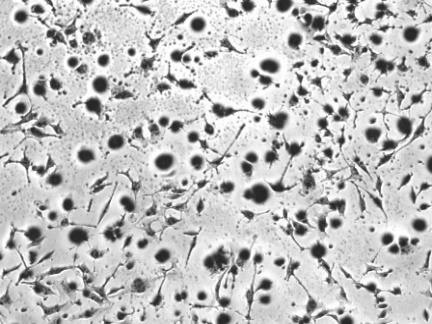**  **500 µM** | **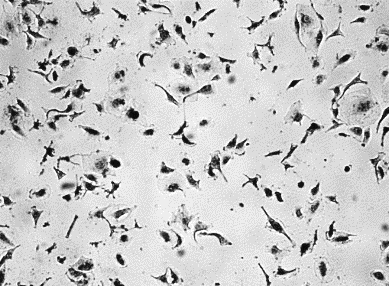**  **500 µM** |
| **GSK346294A**   | **Benzimidazole_N-thiophene series**  **Targets: PLK1, PLK2, PLK3, NEK1, NEK5, NEK9, WNK2, STK10/LOK,** | **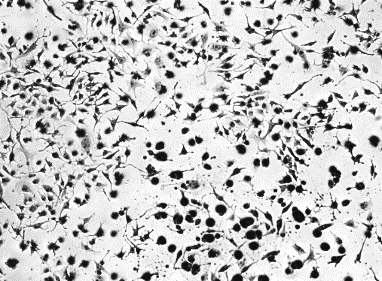**  **500 µM** | **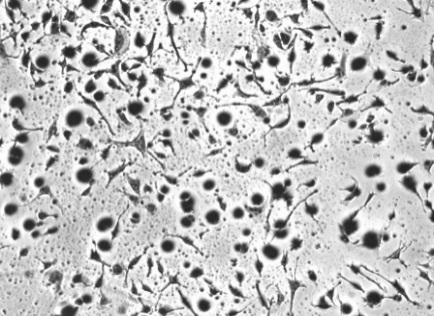**  **500 µM** | **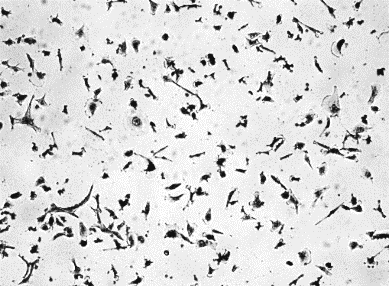**  **500 µM** |
| **GSK350559A**   | **2H-3_pyrimidinyl_pyrazolopyridazine series**  **Targets: DDR1, FGR, YES1, HER4, BMX/ETK, NLK, IGF-1R, INSRR, STK10/LOK** | 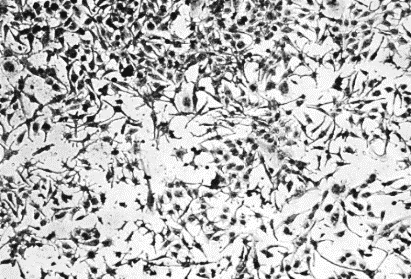  **500 µM** | **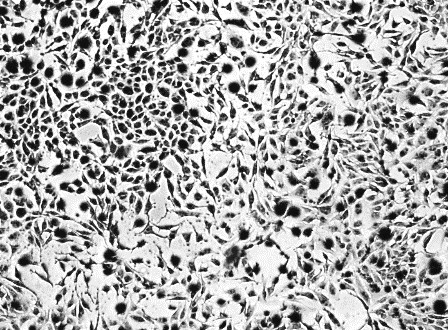**  **500 µM** | **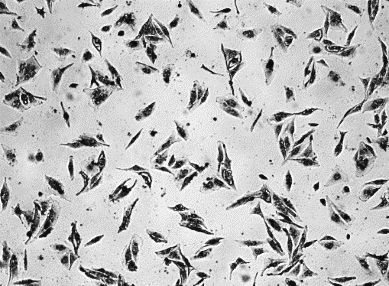**  **500 µM** |
| **GSK198271A**   | **2H-3_pyrimidinyl_pyrazolopyridazine series**  **Targets: DDR1, EGFR, FGR, HER2, YES1, BRK, TAOK2, HCK, HER4, LYN, LCK, CSK, c-Src, STK10/LOK** | **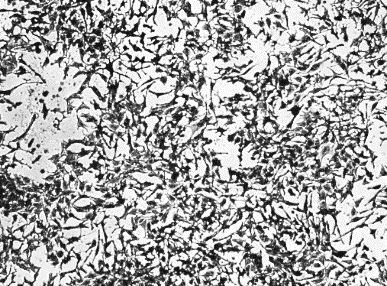**  **500 µM** | **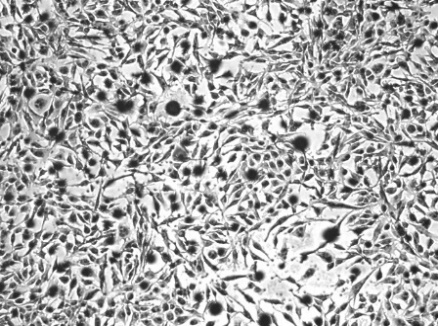**  **500 µM** | **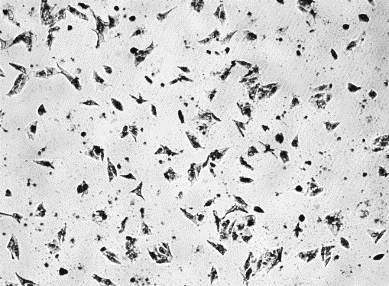**  **500 µM** |
| **GSK1660450B**   |  | 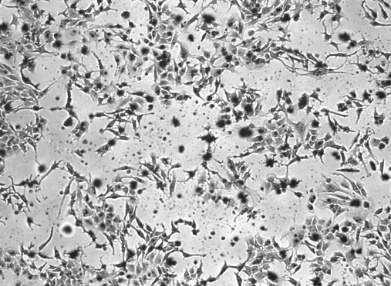  **500 µM** | **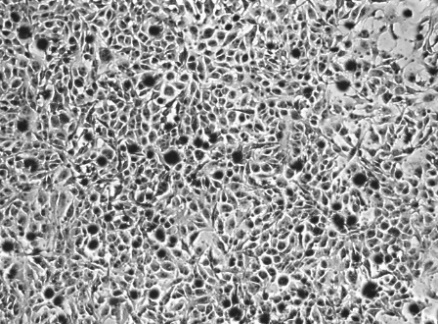**  **500 µM** | **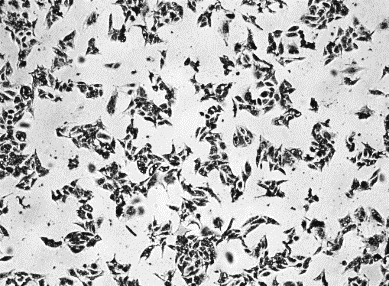**  **500 µM** |
| **GW494610A**   | **4-Aminoquinoline series** | **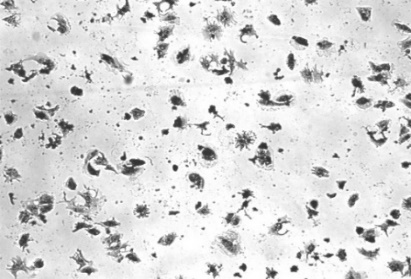**  **500 µM** | **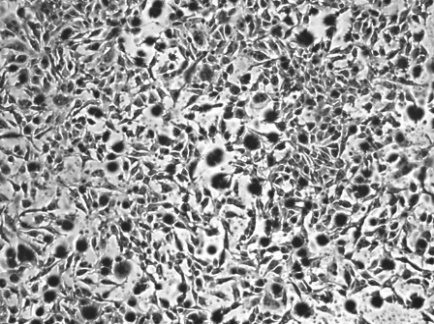**  **500 µM** | **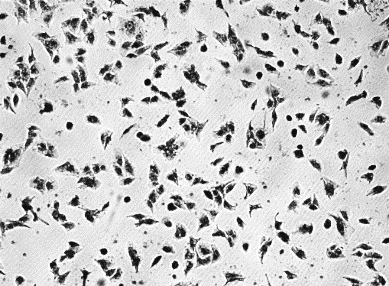**  **500 µM** |
| **GSK1010829B**   |  | **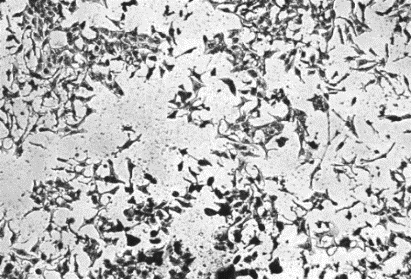**  **500 µM** | **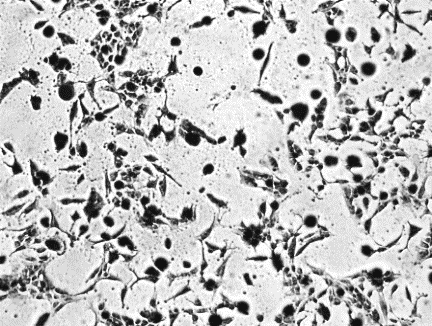**  **500 µM** | **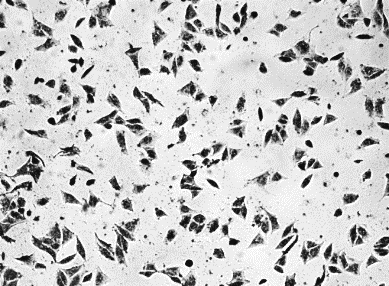**  **500 µM** |
| **GW856804X**   | **Thieno-2-aminopyridine series**  **@ 100 nM, 10 kinases > 50% I**  **Top targets: TSK 82%, MAP4K4 78%, MINK 71%** | **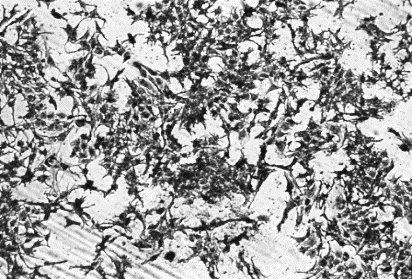**  **500 µM** | **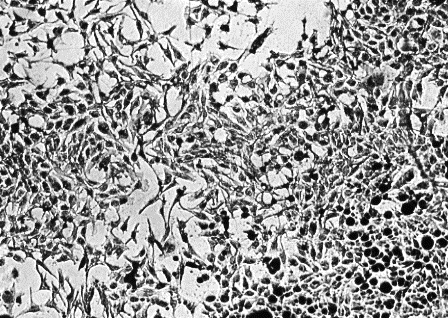**  **500 µM** | **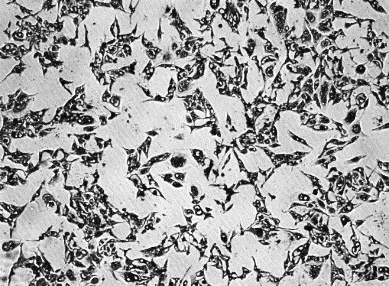**  **500 µM** |
| **GW809897X**   | **2,4-Diamino_pyrimidine series**  **@ 100 nM, 19 kinases > 50% I**  **Top targets: FMS 92%, LOK/STK10 90%, KIT 88%, MET 80%** | **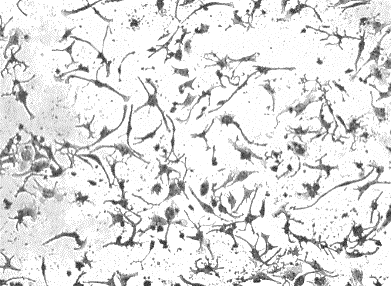**  **500 µM** | **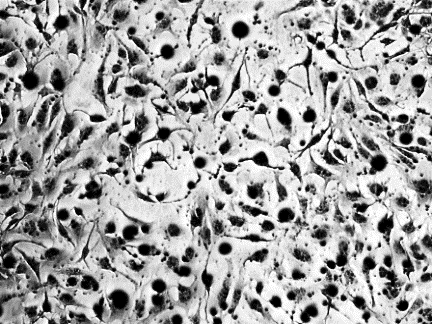**  **500 µM** | **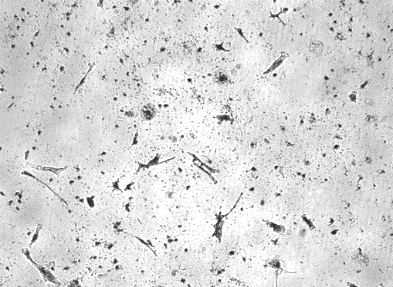**  **500 µM** |
| **GW296115X**   | **Maleimide series** | 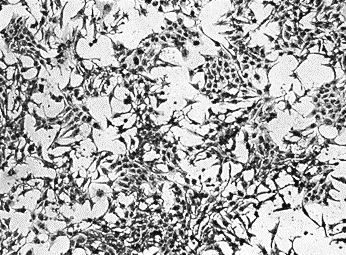  **500 µM** | **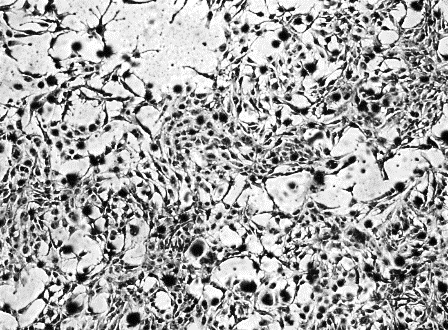**  **500 µM** | **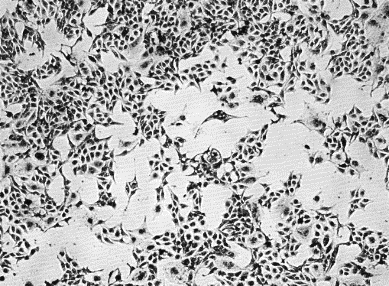**  **500 µM** |
| **GSK1173862A**   | **2,4-Dianilino_pyrrolopyrimidine series**  **@ 100 nM, 21 kinases > 50% I**  **Top targets: INSR 98%, ALK 98%, PYK2 96%, LTK 95%, IGF1R 93%** | **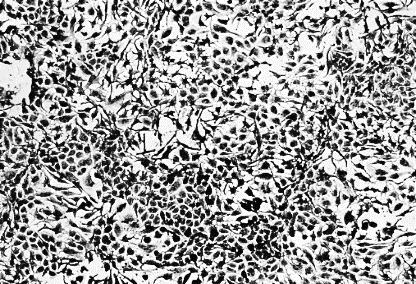**  **500 µM** | **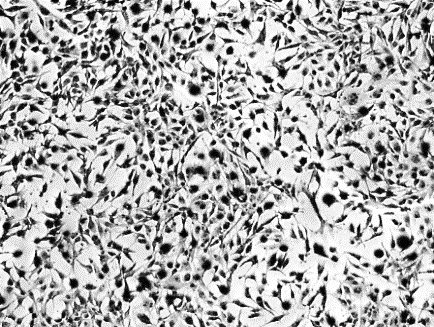**  **500 µM** | **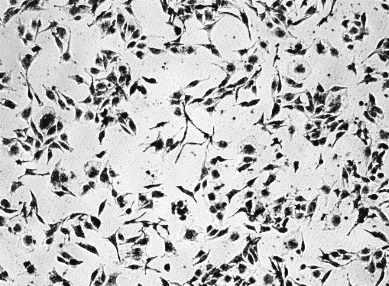**  **500 µM** |
