# Supplementary material for: Novel application of the published kinase inhibitor set to identify therapeutic targets and pathways in triple negative breast cancer subtypes
Source: PLoS One. 2017 Aug 3;12(8):e0177802. doi: 10.1371/journal.pone.0177802 (PMC5542472; doi:10.1371/journal.pone.0177802)
Supplement: S1 Fig — Only GSK809897X appeared to affect the luminal, epithelial non-invasive cell line MCF-7 cell morphologies in addition to triple negative breast cancer cell lines utilized in the cell morphology screen. Images were captured at 100x magnification. (DOCX) [file pone.0177802.s002.docx]

**
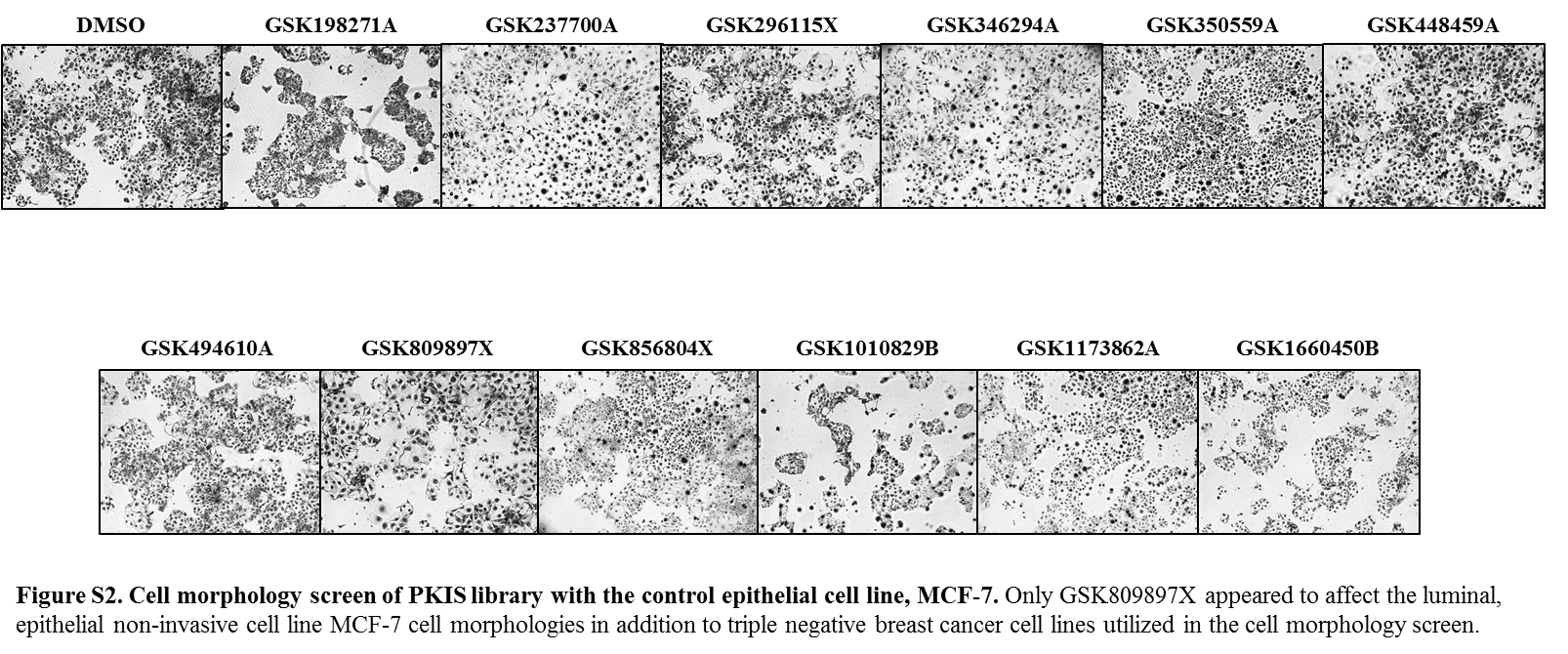
**

**S1 Fig.** **Cell morphology screen of PKIS library with the control epithelial cell line, MCF-7.** Only GSK809897X appeared to affect the luminal, epithelial non-invasive cell line MCF-7 cell morphologies in addition to triple negative breast cancer cell lines utilized in the cell morphology screen. Images were captured at 100x magnification.
