# Supplementary material for: Novel application of the published kinase inhibitor set to identify therapeutic targets and pathways in triple negative breast cancer subtypes
Source: PLoS One. 2017 Aug 3;12(8):e0177802. doi: 10.1371/journal.pone.0177802 (PMC5542472; doi:10.1371/journal.pone.0177802)
Supplement: S2 Fig — Fold change in the mesenchymal protein VIM (S2A) and transcription factors ZEB1 (S2B) was observed after MDA-MB-231 cells were treated with 1 μM of select small molecule inhibitors after 72 hours’ pretreatment. Data was obtained by qRT-PCR and normalized both to actin and the vehicle (DMSO)-treated control designated as 1. N = 3, error bars represent SEM and significantly different * p < 0.05, *** p < 0.001. (DOCX) [file pone.0177802.s003.docx]

**b**

**a**

**S2 Fig.** **PKIS compounds affected mesenchymal gene expression.** Fold change in the mesenchymal protein VIM (S2A) and transcription factors ZEB1 (S2B) was observed after MDA-MB-231 cells were treated with 1 µM of select small molecule inhibitors after 72 hours’ pretreatment. Data was obtained by qRT-PCR and normalized both to actin and the vehicle (DMSO)-treated control designated as 1. N = 3, error bars represent SEM and significantly different * p < 0.05, *** p < 0.001.
