# Supplementary material for: Novel application of the published kinase inhibitor set to identify therapeutic targets and pathways in triple negative breast cancer subtypes
Source: PLoS One. 2017 Aug 3;12(8):e0177802. doi: 10.1371/journal.pone.0177802 (PMC5542472; doi:10.1371/journal.pone.0177802)
Supplement: S3 Fig — E-cadherin molecular weight is 135 kDa (S3A). Rho was utilized to normalize (35 kDa; S3B). (DOCX) [file pone.0177802.s004.docx]

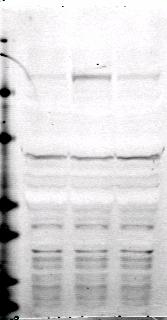

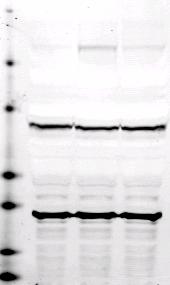


**B.**

**A.**

**S3 Fig.** Example of full-length western blots images of MDA-MB-231 cells treated with DMSO (control, lane 1), GSK198271 (1 µM, 24 hours, lane 2) and GSK350559 (1 µM, 24 hours, lane 3). E-cadherin molecular weight is 135 kDa (S3A). Rho was utilized to normalize (35 kDa; S3B).
