# Supplementary material for: Novel application of the published kinase inhibitor set to identify therapeutic targets and pathways in triple negative breast cancer subtypes
Source: PLoS One. 2017 Aug 3;12(8):e0177802. doi: 10.1371/journal.pone.0177802 (PMC5542472; doi:10.1371/journal.pone.0177802)
Supplement: S4 Fig — Red indicates upregulation, blue indicates downregulation. (DOCX) [file pone.0177802.s005.docx]

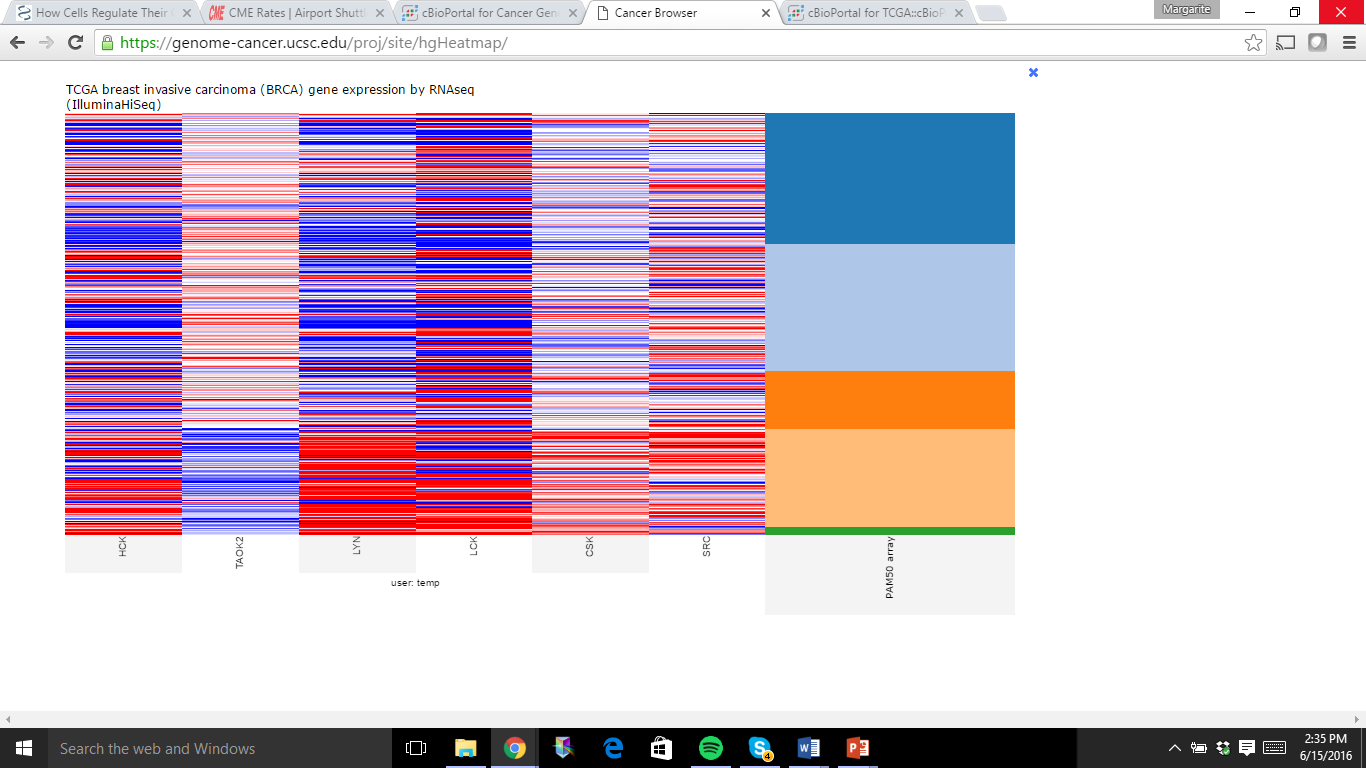


Basal-like

HER2 Enriched

Luminal B

Luminal A

**S4 Fig.** The Cancer Gene Atlas (TCGA) gene expression data shows HCK, TAOK2, LYN, LCK CSK and c-SRC relative gene expression by RNAseq in breast cancer subtypes Luminal A, Luminal B, HER2-enriched and Basal Like. Red indicates upregulation, blue indicates downregulation.
