# Supplementary material for: Novel application of the published kinase inhibitor set to identify therapeutic targets and pathways in triple negative breast cancer subtypes
Source: PLoS One. 2017 Aug 3;12(8):e0177802. doi: 10.1371/journal.pone.0177802 (PMC5542472; doi:10.1371/journal.pone.0177802)
Supplement: S5 Fig — (S5A) High TAOK2 expression is associated with reduced overall survival is a cohort of TNBC-positive patients (n = 68) compared to TNBC-negative patients (n = 526). Hazard ratio was calculated to be 3.06 with a significant p-value of 0.0299. Data provided by the PROGgene database [32,33]. (S5B) TAOK2 and (S5C) HCK are amplified in breast cancers (amplification = red, mutation = green), according to patient data provided by cBioPortal [34,35]. (DOCX) [file pone.0177802.s006.docx]

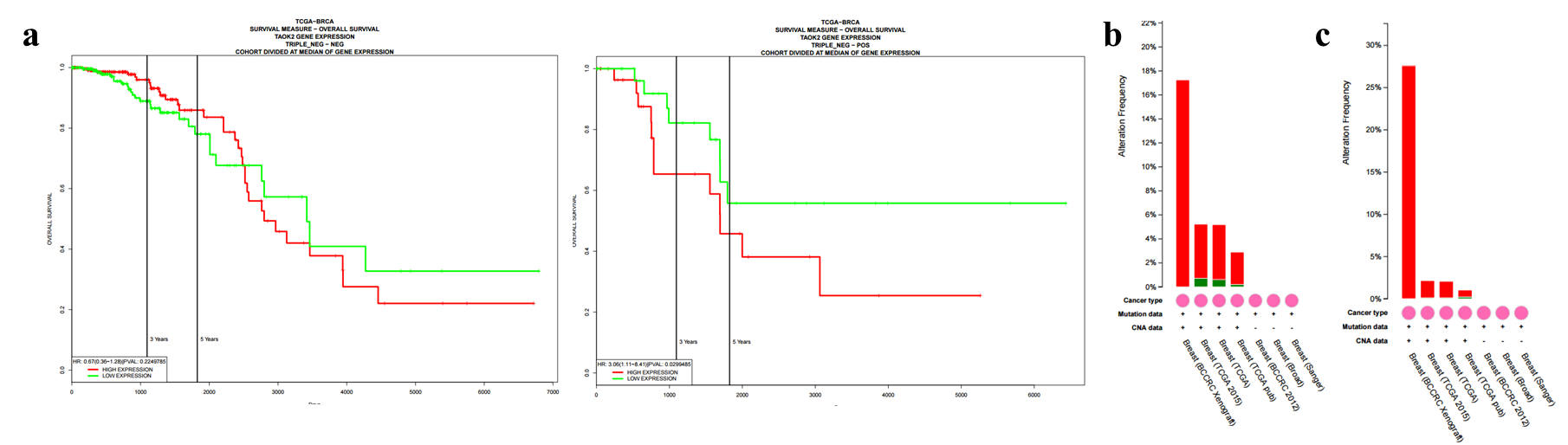


**S5 Fig.** **TAOK2 and HCK expressions in breast cancers.** (S5A) High TAOK2 expression is associated with reduced overall survival is a cohort of TNBC-positive patients (n=68) compared to TNBC-negative patients (n=526). Hazard ratio was calculated to be 3.06 with a significant p-value of 0.0299. Data provided by the PROGgene database [31, 32]. (S5B) TAOK2 and (S5C) HCK are amplified in breast cancers (amplification = red, mutation = green), according to patient data provided by cBioPortal [33, 34].
