# Supplementary material for: Novel application of the published kinase inhibitor set to identify therapeutic targets and pathways in triple negative breast cancer subtypes
Source: PLoS One. 2017 Aug 3;12(8):e0177802. doi: 10.1371/journal.pone.0177802 (PMC5542472; doi:10.1371/journal.pone.0177802)
Supplement: S6 Fig — STK10, and not HCK nor TAOK2, is upregulated in basal B TNBC molecular subtypes. Expression data is provided online and represents the various breast cancer subtypes and was obtained by the Gene expression-based Outcome for Breast cancer Online (GOBO; S6A) [36]. Cell line specific data for STK10, HCK and TAOK2. The various breast cancer cell lines commonly used are shown (S6B). Cell lines are grouped based on the TNBC molecular subtype into which they are categorized: Red indicates basal A subtype, grey indicates basal B subtype and blue indicates luminal subtypes. (DOCX) [file pone.0177802.s007.docx]

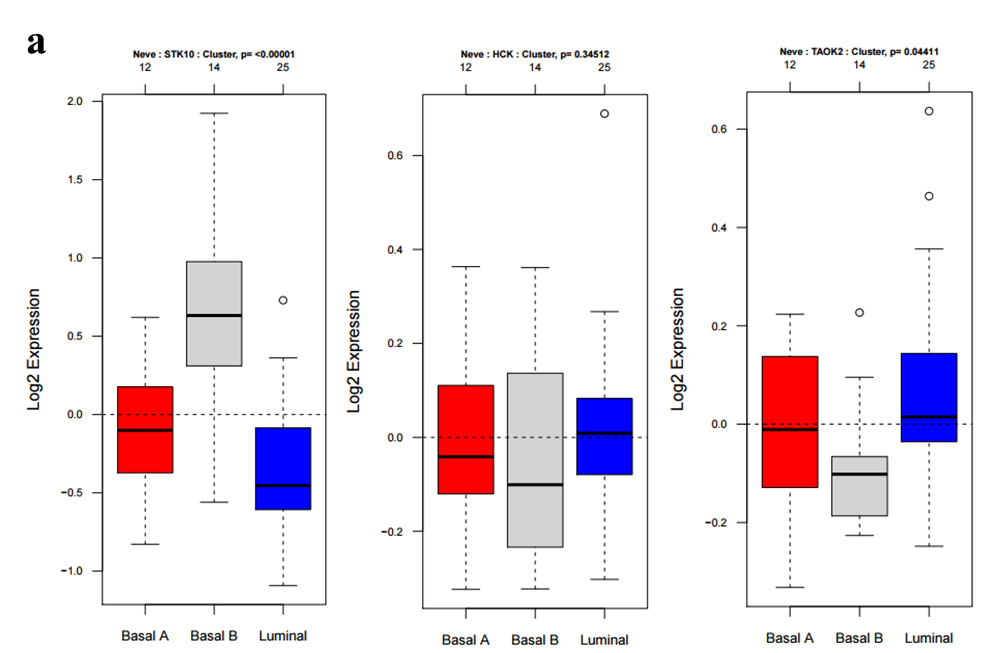


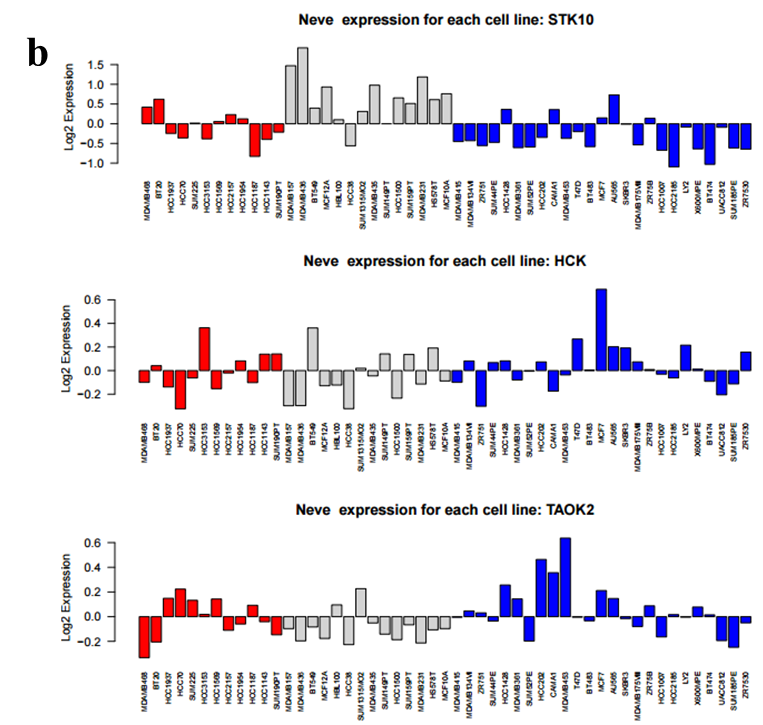


**S6 Fig.** **STK10, HCK and TAOK2 relative expressions in breast cancer cell lines, divided by subtype.** STK10, and not HCK nor TAOK2, is upregulated in basal B TNBC molecular subtypes. Expression data is provided online and represents the various breast cancer subtypes and was obtained by the Gene expression-based Outcome for Breast cancer Online (GOBO; S6A) [35]. Cell line specific data for STK10, HCK and TAOK2. The various breast cancer cell lines commonly used are shown (S6B). Cell lines are grouped based on the TNBC molecular subtype into which they are categorized: Red indicates basal A subtype, grey indicates basal B subtype and blue indicates luminal subtypes.
